# ﻿Checklist of land snail species of Gua Rumbang, Sarawak, Malaysian Borneo (Mollusca, Gastropoda), with a description of a new species, *Diplommatinarumbangensis* sp. nov.

**DOI:** 10.3897/zookeys.1198.116265

**Published:** 2024-04-26

**Authors:** Nurul Syafiqah Nasir, Jie Ying Lee, Mohammad Effendi Marzuki, Jaap J. Vermeulen, Jayasilan Mohd-Azlan, Mohd Zacaery Khalik

**Affiliations:** 1 Faculty of Resource Science and Technology, Universiti Malaysia Sarawak, 94300, Kota Samarahan, Sarawak, Malaysia; 2 Institute of Biodiversity and Environmental Conservation, Universiti Malaysia Sarawak, 94300, Kota Samarahan, Sarawak, Malaysia; 3 JK Art and Science, Lauwerbes 8, 2318 AT, Leiden, Netherlands

**Keywords:** Endemism, habitat types, limestone outcrop, species abundance, species diversity

## Abstract

The current study presents an annotated checklist of the land snail species in the vicinity of the limestone hill of Gua (= cave) Rumbang, an outcrop located at the district of Padawan, Sarawak, Malaysian Borneo. The sampling was conducted at the surrounding areas and near the cave’s entrance. A total of 62 species, involving 19 families and 38 genera, were recorded. Comparison with previous surveys made in the Bau limestone hills revealed similarities with respect to the species-rich families Diplommatinidae and Cyclophoridae, and the genera *Kaliella* and *Diplommatina*, highlighting the regional consistency of the land snail diversity of the Bau-Padawan-Serian cluster. Possibly because of its smaller size, Gua Rumbang is home to two endemic species, while there are eight endemic species in the Bau limestone karsts. This suggests a potential for a significant species diversity within the areas of the limestone ranges that remain to be explored. Nonetheless, the occurrence of endemic species in Gua Rumbang highlights the need to conserve certain areas within the Padawan limestone range since hitherto no protected areas have been proposed in this region. In this checklist, a new species for science is also described, namely, *Diplommatinarumbangensis***sp. nov.**

## ﻿Introduction

Borneo’s karst areas are renowned for their diverse and abundant biodiversity, including species that are endemic to specific sites or regions (Vermeulen and Whitten 1999). The species abundance is mainly caused by the multitude of different ecological niches which typically occur in karst areas, ranging from sun-drenched, bare rock faces to damp, and dark caves (Clements et al. 2006). These ecosystems are characterised by high calcium carbonate deposits and serve as habitat for numerous calcium-dependent organisms, including land snails.

**Figure 1.**
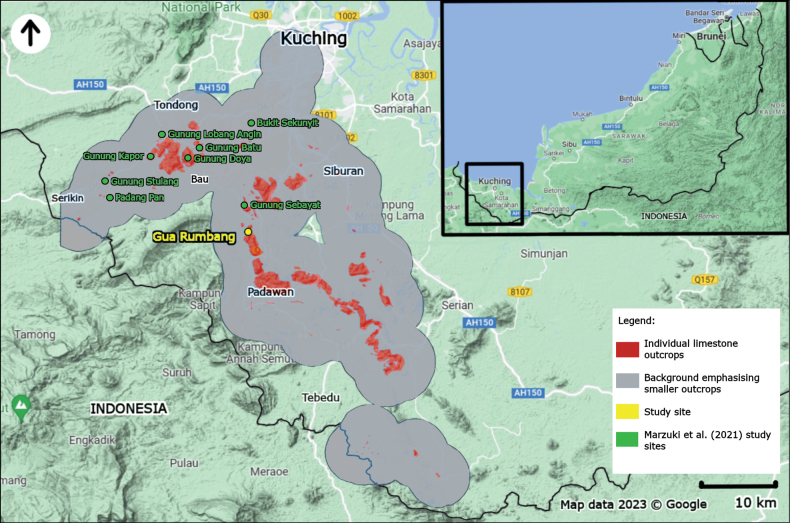
Map showing the location of Gua Rumbang (yellow) on top of an overlay of limestone outcrops in the districts of Kuching, Bau, Siburan, Padawan, Serian, and Tebedu extracted from Liew et al. (2021). The green locations are the hills surveyed by Marzuki et al. (2021). The map highlights individual limestone outcrops with red, whereas grey areas around the limestone outcrops are background to emphasise outcrops that are too small to discern on the map.

Gua (= Cave) Rumbang (1°16.77'N, 110°15.69'E) is located to the north of Gunung Temugan, a limestone outcrop in the Padawan district. Gua Rumbang is part of a long belt of limestone ranges in the south of Kuching division called the ‘Bau-Padawan-Serian’ cluster (see Fig. 1) between the town of Bau in the west, and the Serian district in the east (Liew et al. 2021). This cave has been explored and documented for the first time by Spencer St. John in the 1800s (John 1862). Five species of land snails were described from Gua Rumbang in 1894–1895, namely *Georissaeveretti* E. A. Smith, 1895, *Kaliellarumbangensis* (E. A. Smith, 1895), *Ditropopsiseveretti* (E. A. Smith, 1895), *Plectostomapumilio* (E. A. Smith, 1894a), and *Plectostomaausteni* (E. A. Smith, 1894a). Since then, this limestone hill was not further inventoried. Therefore, this study presents the first checklist of the malacofauna of Gua Rumbang after almost 130 years.

Recently, Marzuki et al. (2021) documented the land snail fauna in the south of Bau district, in the western part of the Kuching limestone ranges. The study listed 122 land snail species including 46 species that are endemic to these ranges. According to Vermeulen (1993, 1994) limestone ranges are areas of endemism, with species occurring restricted to the ranges or parts of them. Despite collecting efforts have focused on the more accessible hills of the ranges, it is probable that some species are endemic to only limited parts of the ranges (Foon et al. 2017; Phung et al. 2018; Foon and Marzuki 2023; Lee et al. 2024). Hence, land snails are a suitable indicator group for limestone biodiversity studies (Vermeulen 2003; Vermeulen and Junau 2007; Liew et al. 2014; Marzuki et al. 2021; Vermeulen and Liew 2022).

In this study, we incorporate the endemic species of these limestone ranges based on literature data, information from the collections at the Zoological Museum of Universiti Malaysia Sarawak (UNIMAS), and the combined knowledge of the authors. Next, we present an annotated checklist of land snail species that were found during our survey. Finally, we describe a new species of land snail of the family Diplommatinidae, namely, *Diplommatinarumbangensis* sp. nov.

## ﻿Materials and methods

### ﻿Land snail sampling and processing

Two separate field surveys were conducted at the surrounding areas near the Gua Rumbang’s entrance (1°16.77'N, 110°15.69'E) on 2 September 2022 and 28 June 2023. Two 20 × 20 m plots were established, and the same plots were sampled for both field surveys. Four persons spent an hour for each plot, totalling 16 person-hours. All living snails and empty shells were collected during the search. This includes sifting through leaf litter, scanning the surface of limestone rocks, wood logs, and the surrounding karst vegetation. The microhabitats where the land snails were found were characterized with respect to their leaf litter, limestone, and vegetation (see Table 1). Leaf litter is the surface litter of the outermost layer of the ground. Limestone rocks refers to the vertical and horizontal wall surfaces, pockets, and cervices. Vegetation refers to the leaf surface, tree trunks and vines. Approximately five litres of soil collected from the area were dried before micro snails and shells were extracted by floatation and left to dry. Living specimens were stored in sample vials containing 70% ethanol. Empty shells were cleaned and dried prior to storage in the museum collection. Specimens were identified by their shell morphology using the literature of Bornean land snails (Vermeulen 1991, 1993, 1994, 1996; Khalik et al. 2019; Marzuki et al. 2021; Vermeulen and Liew 2022). The material was deposited at the Zoological Museum UNIMAS (**ZMU**), with duplicate specimens deposited in the private collection of the third author (**ME**).

**Table 1.** List of living snails collected in Gua Rumbang and their habitat types.

Family / Species	Number of individuals	Relative abundance (%)	Habitat types		
			Leaf litter	Vegetation	Limestone
**Alycaeidae**					
*Pincernaglobosa*	59	2	–	59	–
*Stomacosmethishosei*	492	16.7	3	–	489
**Ariophantidae**					
*Rahulararicostulata*	21	0.7	21	–	–
*Macrochlamyssanctijohni*	1	0.03	1	–	–
*Macrochlamysinfans*	2	0.07	1	1	–
*Vitrinulaglutinosa*	2	0.07	–	2	–
**Camaenidae**					
*Amphidromusangulatus*	1	0.03	–	1	–
*Amphidromusepidemiae*	2	0.07	–	2	–
**Chronidae**					
*Kaliellabusauensis*	28	0.95	–	28	–
*Kaliellabarrakporensis*	73	2.5	–	73	–
*Kaliellacalculosa*	4	0.1	–	4	–
*Kaliellamicroconus*	67	2.3	–	67	–
*Kaliellarumbangensis*	585	19.9	–	552	33
*Kaliellascandens*	3	0.1	–	3	–
*Exrhysotabrookei*	1	0.03	–	–	1
**Cyclophoridae**					
*Cyclophorusperdixborneensis*	2	0.07	2	–	–
*Craspedotropisborneensis*	176	6	173	–	3
*Japoniabarbata*	1	0.03	1	–	–
*Japoniaborneensis*	3	0.1	3	–	–
*Japoniamundyana*	1	0.03	–	1	–
*Opisthoporusbiciliatus*	2	0.07	1	–	1
**Diapheridae**					
*Platycochliumsarawakense*	270	9.2	270	–	–
**Diplommatinidae**					
*Diplommatinabaritensis*	111	3.8	111	–	–
*Diplommatinaconcinna*	2	0.07	2	–	–
*Diplommatinamaduanamaduana*	6	0.2	6	–	–
*Diplommatinaadversa*	5	0.2	5	–	–
*Plectostomaausteni*	34	1.2	–	–	34
*Plectostomaanisopterum*	120	4.1	–	–	120
*Plectostomapumilio*	670	22.6	–	–	670
*Opisthostomabrachyacrumlambii*	44	1.5	44	–	–
*Opisthostomatridens*	6	0.2	–	–	6
**Dyakiidae**					
*Dyakiasubdebilis*	4	0.1	–	3	1
*Rhinocochlisnasuta*	2	0.07	–	2	–
**Helicarionidae**					
*Helicariondyakanum*	2	0.07	–	2	–
**Hydrocenidae**					
*Georissaeveretti*	17	0.6	–	–	17
**Punctidae**					
*Paralaomasarawakensis*	96	3.3	96	–	–
**Valloniidae**					
*Pupisomadioscoricola*	33	1.1	–	33	–
**Total**	2,948		740	833	1,375

### ﻿Imaging and scanning electron microscopy

A representative shell of each species was selected for imaging. A set of stacked images were taken using a Nikon DSLR with CaptureOne 15.0.0 software. Then, the composite images were generated in Helicon 8.2.0 software. The images were edited using Adobe Photoshop 24.1 and GIMP 2.10.34 software. Scanning electron microscopy was used to obtain detailed images of *Diplommatinarumbangensis* sp. nov. To this end shells of the new species were coated with platinum.

### ﻿Land snails diversity and endemism

The species diversity observed at Gua Rumbang was compared with land snail diversity data from elsewhere in Sarawak, including Bau (Marzuki et al. 2021), and limestone hills outside of western Sarawak namely Bukit (= Hill) Sarang (Vermeulen and Junau 2007), Niah National Park (NP), and Gunung (= Mountain) Mulu National Park (NP) (Vermeulen 2003). These localities are isolated and far from the limestone ranges of western Sarawak, since Niah NP and Gunung Mulu NP are in the east, while Bukit Sarang is located in central Sarawak. We evaluated species diversity by considering two factors: (1) species richness, i.e. the total number of species per locality, and (2) the number of unique species, i.e. the number of species that occur only in one of the surveyed limestone hills. These species are for the time being considered unique, even if some of them may turn up in other limestone hills that are yet-to-be surveyed (Foon et al. 2017). Representative land snails sampled during the surveys are shown in Figs 4–24. The following abbreviations are used in the text:

**SH** Shell height

**SW** Shell width

**ME** Marzuki Effendi

**NP** National Park

**UNIMAS** Universiti Malaysia Sarawak

**ZMU** Zoological Museum UNIMAS

## ﻿Results and discussion

### ﻿Land snail diversity and endemism

A total of 5,221 individuals were obtained from surveys done at Gua Rumbang comprising 62 species of land snails belonging to 38 genera and 19 families (see Suppl. material 1). The family of Diplommatinidae was the most species-rich family, with 11 species. This was followed by Cyclophoridae with ten species. The most diverse genera were *Diplommatina* and *Kaliella* with six species each.

Our study targeted the land snails of Gua Rumbang in Padawan, which is a hill in the central part of the Bau-Padawan-Serian limestone range of western Sarawak. The malacofauna of the Padawan limestone ranges remains largely to be explored. When comparing with the malacofauna survey conducted by Marzuki et al. (2021) in Bau, the species compositions are similar in terms of the most diverse families (i.e., Diplommatinidae and Cyclophoridae) and genera (i.e., *Kaliella* and *Diplommatina*). Marzuki et al. (2021) recorded eight endemic land snail species, whereas this study found two endemic species in Gua Rumbang (Table 2). This probably reflects the smaller sampling area at Gua Rumbang compared to the more extensive sampling area covering eight limestone hills in the Bau limestone range. Among these eight limestone hills, Gunung Kapor had the highest number of species (*n* = 11) that did not overlap with the other limestone hills. Of these 11 species, only two species are endemic to Gunung Kapor.

**Table 2.** List of limestone hills with the number of species richness and endemic species in Sarawak.

Limestone hill	Area (km²)	Species richness	Endemic species	Reference
Niah National Park	9	108	38	Vermeulen 2003
Gunung Mulu National Park	80	97	33	Vermeulen 2003
Bukit Sarang	0.3	83	26	Vermeulen and Junau 2007
Bau limestone ranges				
Gunung Kapor	0.08	91	2	Marzuki et al. 2021
Gunung Batu	0.06	83	3	
Gunung Doya	0.09	78	1	
Gunung Lobang Angin	0.07	50	2	
Padang Pan	0.02	25	0	
Bukit Sekunyit	0.07	17	0	
Gunung Sebayat	0.03	14	0	
Gunung Stulang	0.06	12	0	
Gua Rumbang	0.04	62	2	Current study

When compared with other limestone karsts in Sarawak beyond the Bau-Padawan-Serian cluster, Gua Rumbang possesses the lowest species richness and number of endemic species (Table 2). Both Niah NP and Gunung Mulu NP have a higher species richness and more endemic species than Gua Rumbang. Also, this may be explained by the larger areas of both these national parks compared to Gua Rumbang. In contrast, Bukit Sarang is home to a large number of endemic species despite its relatively small area (0.3 km^2^). The high endemism of land snails in Bukit Sarang may be due to its geographic isolation from nearby limestone ranges, such as Ulu Kakus, which is 60 km away.

Based on these comparisons, two species are endemic to Gua Rumbang, but when considered together with the Bau-Padawan-Serian cluster, the number increases to 50 endemic species. No fewer than 80 species from the Bau-Padawan-Serian cluster can be found beyond the southwestern Sarawak limestone clusters. There are also 37 species from Gua Rumbang that have a wider distribution beyond the borders of Sarawak. However, these comparisons should be interpreted with some reservation since each study used different sampling methods, so that their degrees of coverage and completeness may differ. Consequently, the assessments and explanations regarding species richness and endemism are only preliminary and could change if a standardized sampling regime is applied (sensu Clements et al. 2008; Liew et al. 2008; Foon et al. 2017).

The presence of species that are endemic to only a small part of the range complicates effective biodiversity conservation of the Bau-Padawan-Serian limestone ranges. Based on Marzuki et al. (2021), four out of eight surveyed limestone hills in Bau had endemic species (Table 2). This could indicate a high species diversity for the unexplored parts of the Bau-Padawan-Serian limestone range. Hence, it may be necessary to conserve several parts of the ranges to safeguard a representative selection of the limestone biodiversity. Gua Rumbang is a part of Gunung Temugan, with an approximate size of 6.3656 km^2^ (Liew et al. 2021) and no protected areas have been proposed in this vicinity. The selection of the areas should be based on studies of the fauna composition of the whole of the Bau-Padawan-Serian limestone ranges. In this context, land snails are a suitable indicator group for such studies, as their abundance and species composition can reflect the impact of habitat fragmentation and disturbance (Nekola 2012; Douglas et al. 2013; Dhiman et al. 2020; Lee et al. 2024).

## ﻿Systematics

**Class Gastropoda Cuvier, 1795**,

**Subclass Caenogastropoda Cox, 1960**

**Family Diplommatinidae L. Pfeiffer, 1856**

**Genus *Diplommatina* Benson, 1849**

### ﻿ Diplommatina rumbangensis sp. nov.

Taxon classificationAnimaliaGastropodaCaenogastropoda

E51B780E-9C8C-5A10-A00E-73204F39FAD2

https://zoobank.org/C34336B3-6421-49FD-84AC-154E8A1C4691

Figs 2A–F , 8E

#### Type material examined.

***Holotype*.** Malaysia • (SH = 5.25 mm, SW = 2.52 mm); Sarawak, Gua Rumbang, near Kampung Semadang, along Sarawak Kanan River, ~ 11 miles Southwest Padawan, Kuching Division; 1°16.77'N, 110°15.69'E; 2 Sep. 2022; N.S. Nasir and M. E. Marzuki leg.; MZU.MOL.22.132. ***Paratypes*.** Malaysia • 4 ex. (SH = 5.1 mm, SW = 2.7 mm; SH = 5.06 mm, SW = 2.5 mm; SH = 5.37 mm, SW = 2.58 mm; SH = 5.01 mm, SW = 2.65 mm); same data as holotype; MZU.MOL.22.491, ME 14983, ME 15021. Both holotype and paratypes were deposited in Zoological Museum UNIMAS (ZMU) and additional paratypes in the private collection of the third author.

**Figure 2.**
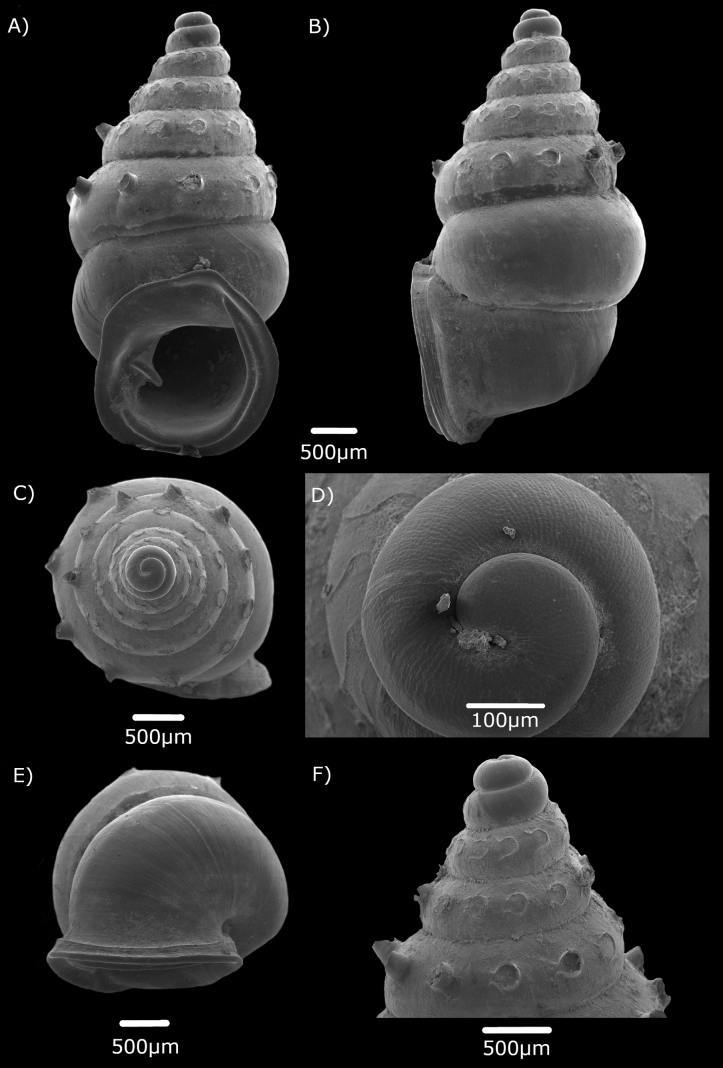
Scanning Electron Microscope images of *Diplommatinarumbangensis* sp. nov. **A–F** Paratypes (ME 14471). **A** apertural view **B** side view **C** apical view **D** enlargement of apical view showing the apex with radial ribs **E** umbilical view **F** top whorls view showing the tubular projection and semi-circular scars.

#### Description.

Shell dextral, fusiform to moderately conical, reddish orange, shining and translucent, with the penultimate whorl widest, convex, well rounded. Suture impressed. Protoconch with 1½ whorls, punctate with small pits, without radial and spiral lines. Constriction nearly level with the edge between the parietal and columellar side of the peristome, with two parietales, two upper longitudinal palatales which are not covered by the peristome on the outer surface of the shell, one transversal palatalis and one columellaris. The columellaris positioned at the start of the constriction together with the longitudinal palatales. Tuba approximately ¾ whorl. ***Sculpture***: Radial ribs on the top whorls only, widely spaced, inconspicuous, but halfway the whorl with an almost tubular projection, in adults sometimes abraded to a semi-circular scar. Spiral striation inconspicuous, on top whorls only. ***Aperture***: Hardly tilted regarding the coiling axis; columellaris distinct, directed downwards. Peristome double, expanding; palatal side hardly sinuous, without edge; basal side with an edge; basal edge hardly sinuous, rounded; inner peristome somewhat expanding beyond the outer, with a palatal lip, free and erect on the columellar side, expanding on the parietal side. ***Umbilicus***: Open, narrow. ***Dimensions***: Height 5.01–5.37 mm; width 2.5–2.7 mm; number of whorls 6¾–7; height and width aperture 1.91–2.05 mm; 2.05–2.09 mm.

#### Differential diagnosis.

**Figure 3.**
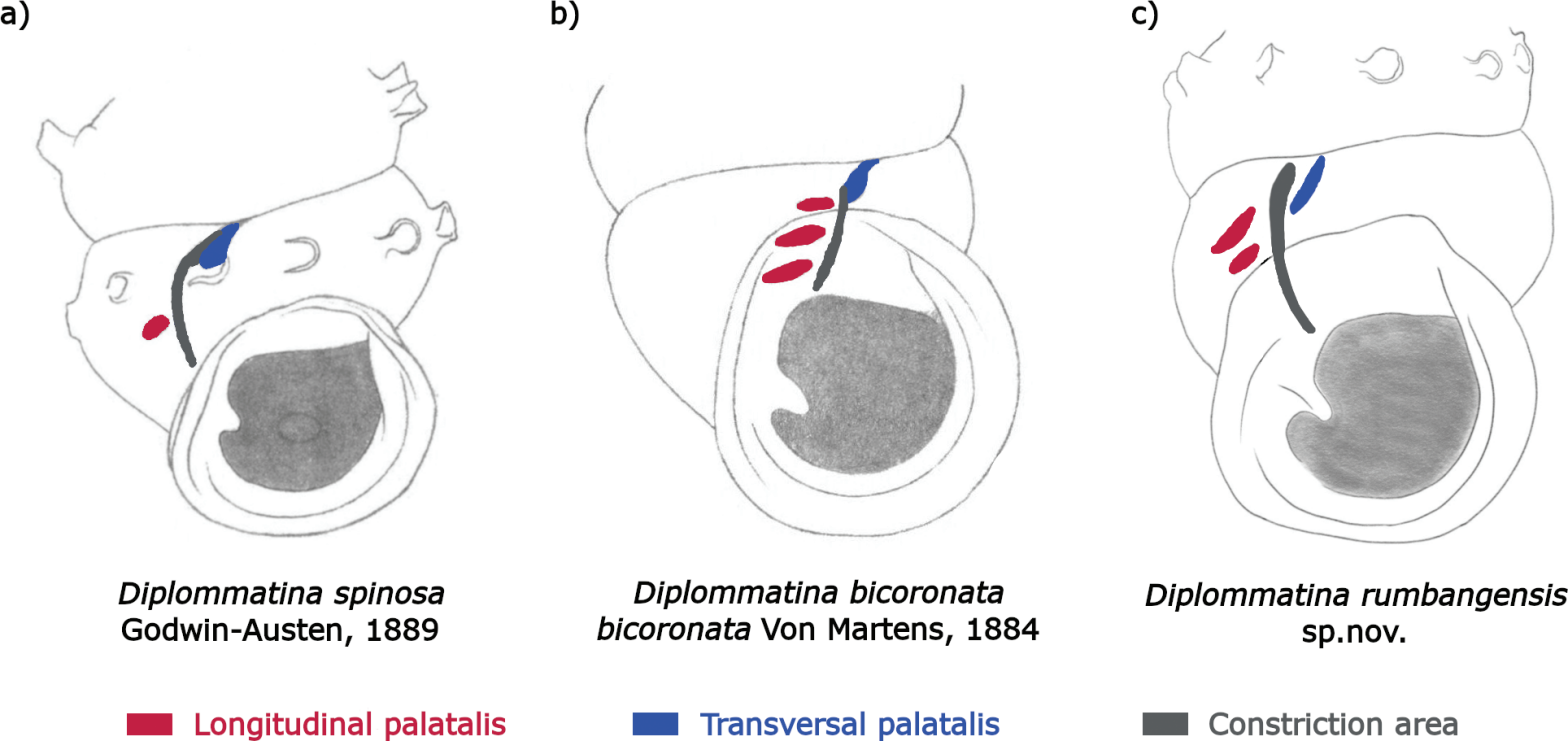
Sketch of *Diplommatinaspinosa* and *Diplommatinabicoronatabicoronata* extracted from Vermeulen (1993), and *Diplommatinarumbangensis* sp. nov. showing the position of the longitudinal palatalis (red), the transversal palatalis (blue), and the constriction area (grey).

*Diplommatinarumbangensis* sp. nov. has two distinct upper longitudinal palatales at the shell constriction that are not covered by the peristome on the outer surface of the shell (Fig. 3). This differs from *Diplommatinaspinosa* Godwin-Austen, 1889 which has one longitudinal palatalis only. Additionally, *D.spinosa* exhibits a tubular projection that extends to the penultimate whorl, whereas in *D.rumbangensis* sp. nov., this projection only reaches halfway through the ultimate whorl. *Diplommatinaspinosa* is distributed in the Kuching and Serian divisions. It also differs from *Diplommatinabicoronatabicoronata* von Martens, 1884 which is endemic to Kalimantan, Indonesia by having two or three longitudinal palatales with two of them covered by the peristome. *Diplommatinabicoronatabicoronata* also has radial ribs near the suture of the whorls which is absent in *D.rumbangensis* sp. nov. This latter species can be distinguished from *Diplommatinaaurisdiaboli* Vermeulen, 1993 by the absence of palatal lip expanding up the suture of the previous whorl.

#### Etymology.

The epithet *rumbangensis* refers to the type locality Gua Rumbang.

#### Geographic distribution and habitat.

*Diplommatinarumbangensis* sp. nov. is known from the type locality only. The living animals were not observed.

#### Remarks.

The spine or tubular projection of the shells of *Diplommatinarumbangensis* sp. nov. are mostly broken, leaving semi-circular scars.

##### ﻿Checklist

**Class Gastropoda Cuvier, 1795**,

**Subclass Caenogastropoda Cox, 1960**

**Family Alycaeidae W. T. Blanford, 1864**

***Chamalycaeusspecus* (Godwin-Austen, 1889)**

Fig. 4A

**Figure 4.**
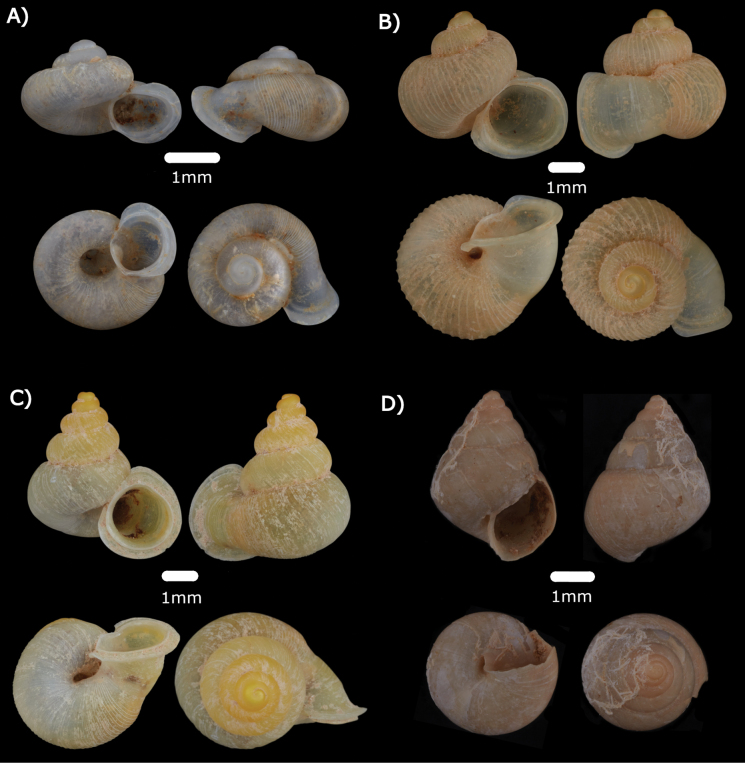
**A***Chamalycaeusspecus* (Godwin-Austen, 1889) ME 14470 **B***Pincernaglobosa* (H. Adams, 1871) ME 14468 **C***Stomacosmethishosei* (Godwin-Austen, 1889) ME 14469 **D***Solenomphalascalaris* (Heude, 1882) ME 14512.

**Type locality.** “In limestone caves at Jambusan, Borneo”.

**Material examined.** Malaysia • Sarawak, Padawan, Gua Rumbang; 1°16.77'N, 110°15.69'E; 2 Apr. 2016–28 Jun. 2023; N.S. Nasir, M.E Marzuki, J.Y. Lee, and M.Z Khalik leg.; ME 14470, ME 14982, ME 15020, MZU.MOL.16.116.

**Distribution.** Widespread in Borneo. Endemic to Borneo (Marzuki et al. 2021; Vermeulen and Liew 2022).

**Remarks.** Among leaf-litter and plant debris at the base of limestone cliffs. Only empty shells were found.

***Pincernaglobosa* (H. Adams, 1871)**

Figs 4B, 19A

**Type locality.** “Busan, near Sarawak, Borneo”.

**Material examined.** Malaysia • Sarawak, Padawan, Gua Rumbang; 1°16.77'N, 110°15.69'E; 2 Apr. 2016–28 Jun. 2023; N.S. Nasir, M.E Marzuki, J.Y. Lee, and M.Z Khalik leg.; ME 14468, ME 15018, MZU.MOL.16.109, MZU.MOL.22.135, MZU.MOL.23.139.

**Distribution.** Scattered localities between Bau and Serian-Padawan limestone hills in western Sarawak to Niah, further to the north. Also found in Sabah and West Kalimantan. Endemic to Borneo (Marzuki et al. 2021; Vermeulen and Liew 2022).

**Remarks.** Living snails were observed foraging on leaf surface of trees at the base of limestone cliffs.

***Stomacosmethishosei* (Godwin-Austen, 1889)**

Figs 4C, 19B

**Type locality.** “Busan Hills, Borneo”.

**Material examined.** Malaysia • Sarawak, Padawan, Gua Rumbang; 1°16.77'N, 110°15.69'E; 2 Apr. 2016–28 Jun. 2023; N.S. Nasir, M.E Marzuki, J.Y. Lee, and M.Z Khalik leg.; ME 14469, ME 14981, ME 15019, MZU.MOL.16.108, MZU.MOL.22.136

**Distribution.** Scattered localities between Bau and Serian-Padawan limestone hills in western Sarawak. Endemic to western Sarawak (Marzuki et al. 2021).

**Remarks.** Living snails found on wet limestone surfaces covered with mosses. Empty shells were found among leaf litter at the base of limestone hills.

**Family Assimineidae H. Adams & A. Adams, 1856**

***Solenomphalascalaris* (Heude, 1882)**

Fig. 4D

**Type locality.** “Ad parietes humidos in civitate Chang-hai sat copiosa” [= Shanghai, China].

**Material examined.** Malaysia • Sarawak, Padawan, Gua Rumbang; 1°16.77'N, 110°15.69'E; 2 Sep. 2022; N.S. Nasir, M.E Marzuki, J.Y. Lee, and M.Z Khalik leg.; ME 14512.

**Distribution.** Widely distributed in Borneo. Also found in China and Peninsular Malaysia (Marzuki et al. 2021; Vermeulen and Liew 2022).

**Remarks.** Only empty shells were found. An introduced species. Records show that it occurs in the damp areas around human settlements (Chan 1997).

**Family Cyclophoridae Gray, 1847**

***Craspedotropisborneensis* (Godwin-Austen, 1889)**

Figs 5A, 19C

**Figure 5.**
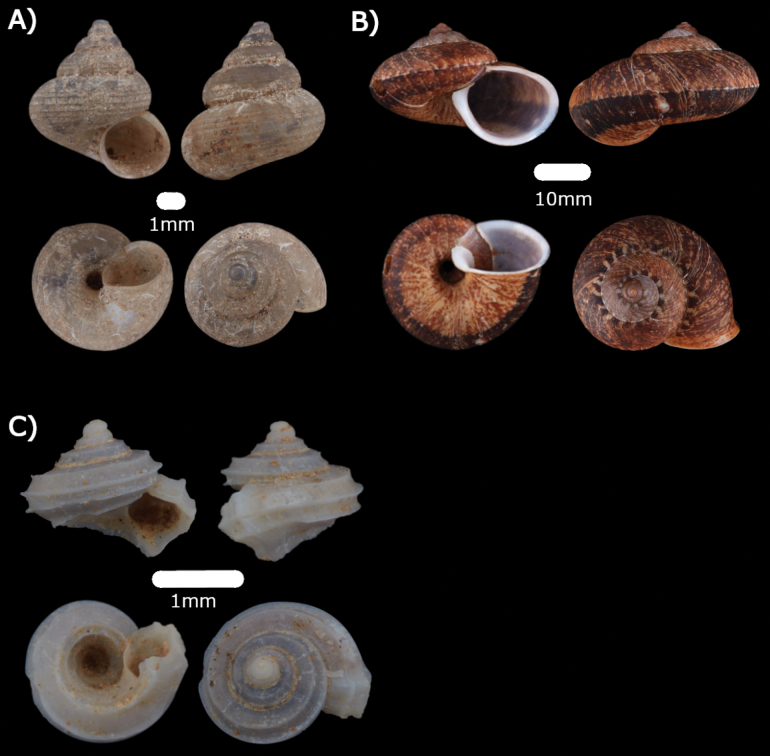
**A***Craspedotropisborneensis* (Godwin-Austen, 1889) MZU.MOL.22.137 **B***Cyclophorusperdixborneensis* (Metcalfe, 1852) MZU.MOL.22.184 **C***Ditropopsiseveretti* (E. A Smith, 1895) ME 14467.

**Type locality.** “Busan Hills, Borneo” [= Jambusan Hills, Sarawak].

**Material examined.** Malaysia • Sarawak, Padawan, Gua Rumbang; 1°16.77'N, 110°15.69'E; 2 Sep. 2022–28 Jun. 2023; N.S. Nasir, M.E Marzuki, J.Y. Lee, and M.Z Khalik leg.; ME 14980, ME 15016. MZU.MOL.22.137, MZU.MOL.23.141.

**Distribution.** Scattered localities between Bau and Serian-Padawan limestone hills in western Sarawak. Endemic to western Sarawak.

**Remarks.** Living snails were found foraging on limestone surfaces and among leaf-litter and topsoil at the base of limestone cliffs. Living individuals of this species are always covered by dirt, which makes it difficult to be spotted.

***Cyclophorusperdixborneensis* (Metcalfe, 1852)**

Fig. 5B

**Type locality.** “Borneo”.

**Material examined.** Malaysia • Sarawak, Padawan, Gua Rumbang; 1°16.77'N, 110°15.69'E; 2 Sep. 2022–28 Jun. 2023; N.S. Nasir, M.E Marzuki, J.Y. Lee, and M.Z Khalik leg.; ME 14461, ME 15010, MZU.MOL.22.184.

**Distribution.** Widely distributed in Borneo and Sarawak but rare in Sabah (Marzuki et al. 2021; Vermeulen and Liew 2022). Also found in West Malaysia (Stoliczka 1872; Morgan 1885).

**Remarks.** Among leaf-litter and plant debris at the base of limestone cliffs. Only empty shells were found.

***Ditropopsiseveretti* (E. A. Smith, 1895)**

Fig. 5C

**Type locality.** “Rumbang, Sarawak” [= Rumbang Hills, Padawan, Sarawak].

**Material examined.** Malaysia • Sarawak, Padawan, Gua Rumbang; 1°16.77'N, 110°15.69'E; 2 Sep. 2022–28 Jun. 2023; N.S. Nasir, M.E Marzuki, J.Y. Lee, and M.Z Khalik leg.; ME 14467, ME 15017.

**Distribution.** Scattered localities between Bau and Serian-Padawan limestone hills in western Sarawak. Endemic to western Sarawak (Marzuki et al. 2021).

**Remarks.** Among leaf-litter and plant debris at the base of limestone cliffs. Only empty shells were found.

***Japoniabarbata* (L. Pfeiffer, 1855)**

Fig. 6A

**Type locality.** “Borneo, Sarawak”.

**Material examined.** Malaysia • Sarawak, Padawan, Gua Rumbang; 1°16.77'N, 110°15.69'E; 2 Sep. 2022–28 Jun. 2023; N.S. Nasir, M.E Marzuki, J.Y. Lee, and M.Z Khalik leg.; ME 14464, ME 15012, MZU.MOL.22.149.

**Distribution.** Scattered localities between Bau and Serian-Padawan limestone hills in western Sarawak to Mukah, also in central Sarawak. Endemic to Sarawak (Marzuki et al. 2021).

**Remarks.** Among leaf-litter and plant debris at the base of limestone cliffs. Only empty shells were found.

***Japoniabauensis* Marzuki, Liew & Mohd-Azlan, 2021**

Fig. 6B

**Figure 6.**
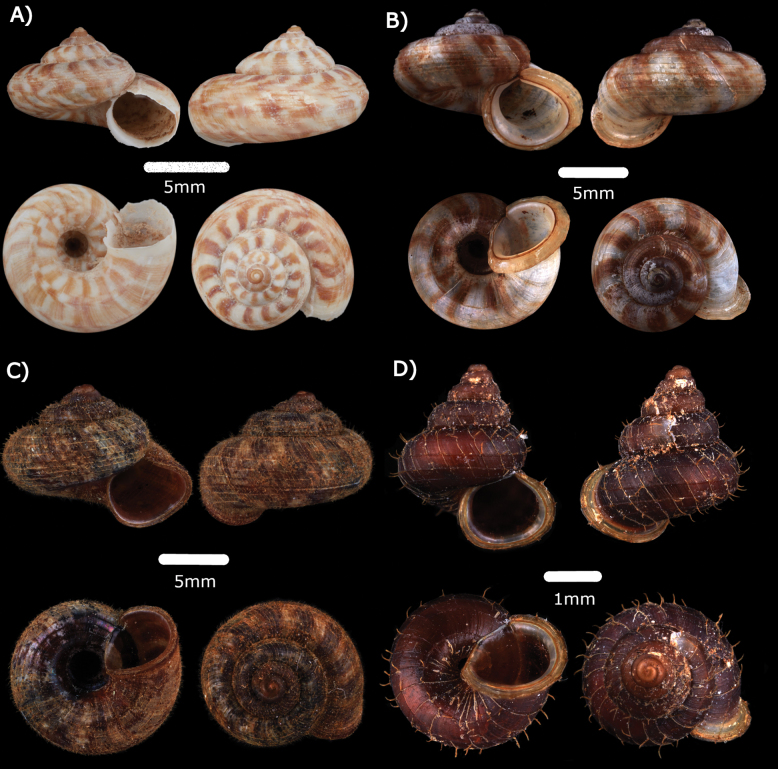
**A***Japoniabarbata* (L. Pfeiffer, 1855) MZU.MOL.22.149 **B***Japoniabauensis* Marzuki, Liew & Mohd-Azlan, 2021 ME 0014465 **C***Japoniaborneensis* (E. A. Smith, 1893) MZU.MOL.22.155 **D***Japoniamundyana* (Godwin-Austen, 1889) MZU.MOL.22.133.

**Type locality.** “Bau and Serian-Padawan limestone hill clusters.”

**Material examined.** Malaysia • Sarawak, Padawan, Gua Rumbang; 1°16.77'N, 110°15.69'E; 2 Sep. 2022–28 Jun. 2023; N.S. Nasir, M.E Marzuki, J.Y. Lee, and M.Z Khalik leg.; ME 14465, ME 15013, MZU.MOL.22.156.

**Distribution.** Scattered localities between Bau and Serian-Padawan limestone hills in Sarawak. Endemic to Sarawak (Marzuki et al. 2021).

**Remarks.** Among leaf-litter and plant debris at the base of limestone cliffs. Only empty shells were found.

***Japoniaborneensis* (E. A. Smith, 1893)**

Figs 6C, 19D

**Type locality.** “Westliches Borneo bei Bengkajang” [= Western Borneo near Bengkayang].

**Material examined.** Malaysia • Sarawak, Padawan, Gua Rumbang; 1°16.77'N, 110°15.69'E; 2 Apr. 2016–28 Jun. 2023; N.S. Nasir, M.E Marzuki, J.Y. Lee, and M.Z Khalik leg.; MZU.MOL.23.138, MZU.MOL.22.155, MZU.MOL.16.110.

**Distribution.** Widely distributed in Borneo (Vermeulen and Liew 2022).

**Remarks.** Living snails were found foraging in leaf-litter and plant debris at the base of limestone cliffs.

***Japoniamundyana* (Godwin-Austen, 1889)**

Fig. 6D

**Type locality.** “Busan Hills, Borneo” [= Jambusan Hills, Bau, Sarawak].

**Material examined.** Malaysia • Sarawak, Padawan, Gua Rumbang; 1°16.77'N, 110°15.69'E; 2 Sep. 2022–28 Jun. 2023; N.S. Nasir, M.E Marzuki, J.Y. Lee, and M.Z Khalik leg.; ME 15014, MZU.MOL.22.133.

**Distribution.** Scattered localities between Bau and Serian-Padawan limestone hills in western Sarawak. Endemic to western Sarawak (Marzuki et al. 2021).

**Remarks.** Living snail was observed foraging on leaf surface of plants at the base of limestone cliffs.

***Leptopomasericatum* (L. Pfeiffer, 1851)**

Fig. 7A

**Figure 7.**
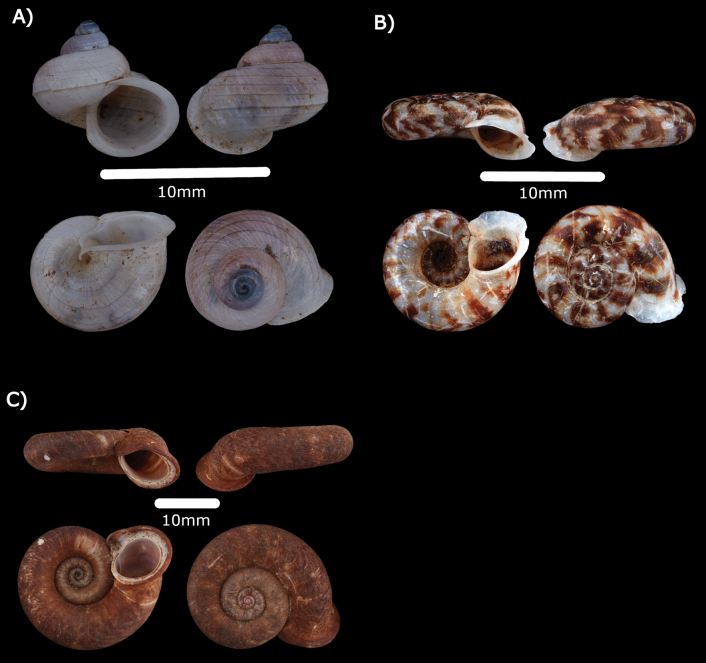
**A***Leptopomasericatum* (L. Pfeiffer, 1851) MZU. MOL.23.135 **B***Opisthoporusbiciliatus* (Mousson, 1849) ME 14463 **C***Pterocyclostenuilabiatus* (Metcalfe, 1852) MZU.MOL.22.129.

**Type locality.** “Borneo”.

**Material examined.** Malaysia • Sarawak, Padawan, Gua Rumbang; 1°16.77'N, 110°15.69'E; 28 Jun. 2023; N.S. Nasir, M.E Marzuki, J.Y. Lee, and M.Z Khalik leg.; ME 15015, MZU.MOL.23.135.

**Distribution.** Widely distributed in Borneo (Marzuki et al. 2021; Vermeulen and Liew 2022).

**Remarks.** Among leaf-litter and plant debris at the base of limestone cliffs. Only empty shells were found.

***Opisthoporusbiciliatus* (Mousson, 1849)**

Figs 7B, 20A

**Type locality.** “Java” [= Borneo (Metcalfe 1852].

**Material examined.** Malaysia • Sarawak, Padawan, Gua Rumbang; 1°16.77'N, 110°15.69'E; 2 Sep. 2022–28 Jun. 2023; N.S. Nasir, M.E Marzuki, J.Y. Lee, and M.Z Khalik leg.; ME 14463, ME 14979, ME 15011, MZU.MOL.22.152.

**Distribution.** Scattered localities between Bau and Serian-Padawan limestone hills in western Sarawak to Mukah, also in central Sarawak. Endemic to Sarawak (Marzuki et al. 2021).

**Remarks.** Among leaf-litter and plant debris at the base of limestone cliffs. Only empty shells were found.

***Pterocyclostenuilabiatus* (Metcalfe, 1852)**

Fig. 7C

**Type locality.** “Borneo”.

**Material examined.** Malaysia • Sarawak, Padawan, Gua Rumbang; 1°16.77'N, 110°15.69'E; 2 Sep. 2022; N.S. Nasir, M.E Marzuki, J.Y. Lee, and M.Z Khalik leg.; ME 14462, ME 14978, MZU.MOL.22.129.

**Distribution.** Widely distributed in Borneo. Endemic to Borneo (Marzuki et al. 2021; Vermeulen and Liew 2022).

**Remarks.** Among leaf-litter and plant debris at the base of limestone cliffs. Only empty shells were found.

**Family Diplommatinidae L. Pfeiffer, 1856**

***Diplommatinaadversa* (H. Adams & A. Adams, 1851)**

Figs 8A, 20B

**Figure 8.**
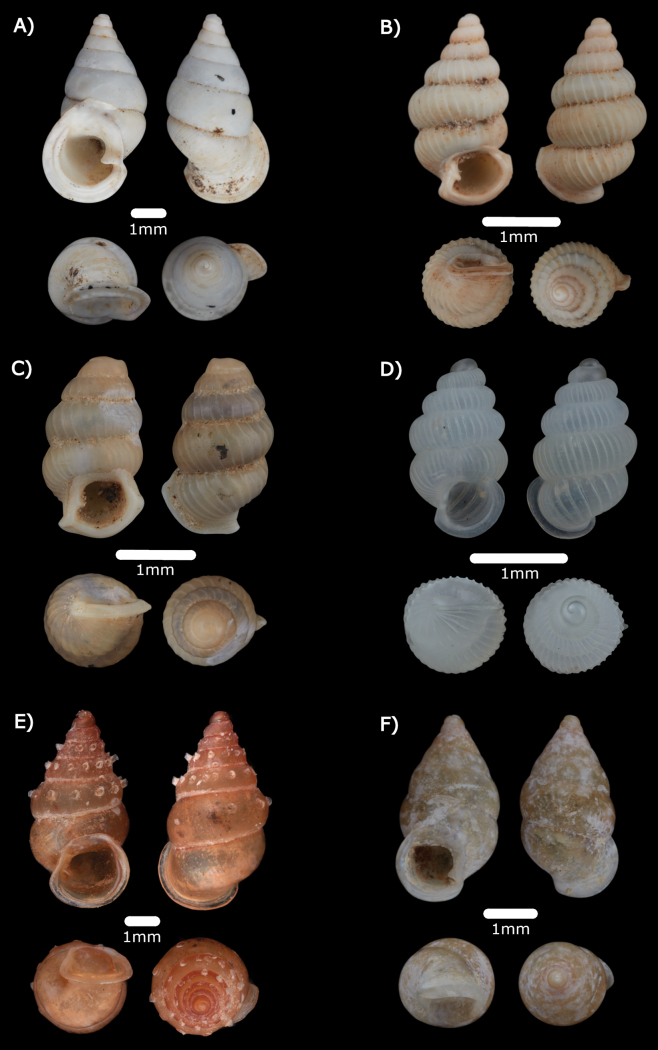
**A***Diplommatinaadversa* (H. Adams & A. Adams, 1851) ME 14476 **B***Diplommatinabaritensis* E. A. Smith, 1893 ME 14472 **C***Diplommatinaconcinna* H. Adams, 1872 ME 14473 **D***Diplommatinamaduanamaduana* Laidlaw, 1949 ME 14474 **E***Diplommatinarumbangensis* sp. nov. holotype MZU.MOL.22.132 **F***Diplommatinasubglabersubisensis* (Vermeulen, 1993) ME 14475.

**Type locality.** “Singapore”.

**Material examined.** Malaysia • Sarawak, Padawan, Gua Rumbang; 1°16.77'N, 110°15.69'E; 2 Sep. 2022–28 Jun. 2023; N.S. Nasir, M.E Marzuki, J.Y. Lee, and M.Z Khalik leg.; ME 14476, MZU.MOL.22.140, MZU.MOL.23.140.

**Distribution.** Scattered localities between Bau and Serian-Padawan limestone hills in western Sarawak. In Sabah found in Ulu Segama only (Marzuki et al. 2021; Vermeulen and Liew 2022). Also found in West Malaysia and Singapore (Laidlaw 1949).

**Remarks.** Living snails were found foraging in leaf-litter and plant debris at the base of limestone cliffs.

***Diplommatinabaritensis* E. A Smith, 1893**

Figs 8B, 20C

**Type locality.** “Barit Mountain, N.W. Borneo”.

**Material examined.** Malaysia • Sarawak, Padawan, Gua Rumbang; 1°16.77'N, 110°15.69'E; 2 Sep. 2022–28 Jun. 2023; N.S. Nasir, M.E Marzuki, J.Y. Lee, and M.Z Khalik leg.; ME 14472, ME 14984, ME 15022, MZU.MOL.22.169, MZU.MOL.23.137.

**Distribution.** Scattered localities between Padawan limestone hills at western Sarawak and Mulu limestone hills in northern Sarawak (Smith 1895; Vermeulen 1993).

**Remarks.** Living snails were found foraging in leaf-litter and plant debris at the base of limestone cliffs.

***Diplommatinaconcinna* H. Adams, 1872**

Fig. 8C

**Type locality.** “Borneo”.

**Material examined.** Malaysia • Sarawak, Padawan, Gua Rumbang; 1°16.77'N, 110°15.69'E; 2 Sep. 2022; N.S. Nasir, M.E Marzuki, J.Y. Lee, and M.Z Khalik leg.; ME 14473, MZU.MOL.22.83.

**Distribution.** Scattered localities between Bau and Serian-Padawan limestone hills in western Sarawak to Niah, in northern Sarawak. Also found in Bunguran, Indonesia (Marzuki et al. 2021) and Singapore (Chan 2020).

**Remarks.** Living snails were found foraging in leaf-litter and plant debris at the base of limestone cliffs.

***Diplommatinamaduanamaduana* Laidlaw, 1949**

Fig. 8D

**Type locality.** “Gua Madu, Kelantan”.

**Material examined.** Malaysia • Sarawak, Padawan, Gua Rumbang; 1°16.77'N, 110°15.69'E; 2 Sep. 2022–28 Jun. 2023; N.S. Nasir, M.E Marzuki, J.Y. Lee, and M.Z Khalik leg.; ME 14474, ME 15023, MZU.MOL.22.190.

**Distribution.** Scattered localities between Bau and Serian-Padawan limestone hills in western Sarawak to Mulu hills in northern Sarawak (Marzuki et al. 2021). Also found in West Malaysia (Laidlaw 1949).

**Remarks.** Living snails were found foraging in leaf-litter and plant debris at the base of limestone cliffs.

***Diplommatinasubglabersubisensis* (Vermeulen, 1993)**

Fig. 8F

**Type locality.** “Sarawak 4^th^ Div.: G. Subis (Batu Niah)”.

**Material examined.** Malaysia • Sarawak, Padawan, Gua Rumbang; 1°16.77'N, 110°15.69'E; 2 Sep. 2022; N.S. Nasir, M.E Marzuki, J.Y. Lee, and M.Z Khalik leg.; ME 14475.

**Distribution.** Scattered localities between Padawan limestone hills in western Sarawak and Niah limestone hills in northern Sarawak (Vermeulen 1993, 1996).

**Remarks.** Among leaf-litter and plant debris at the base of limestone cliffs. Only empty shells were found.

***Opisthostomabrachyacrumlambii* (Vermeulen, 1991)**

Fig. 9A

**Figure 9.**
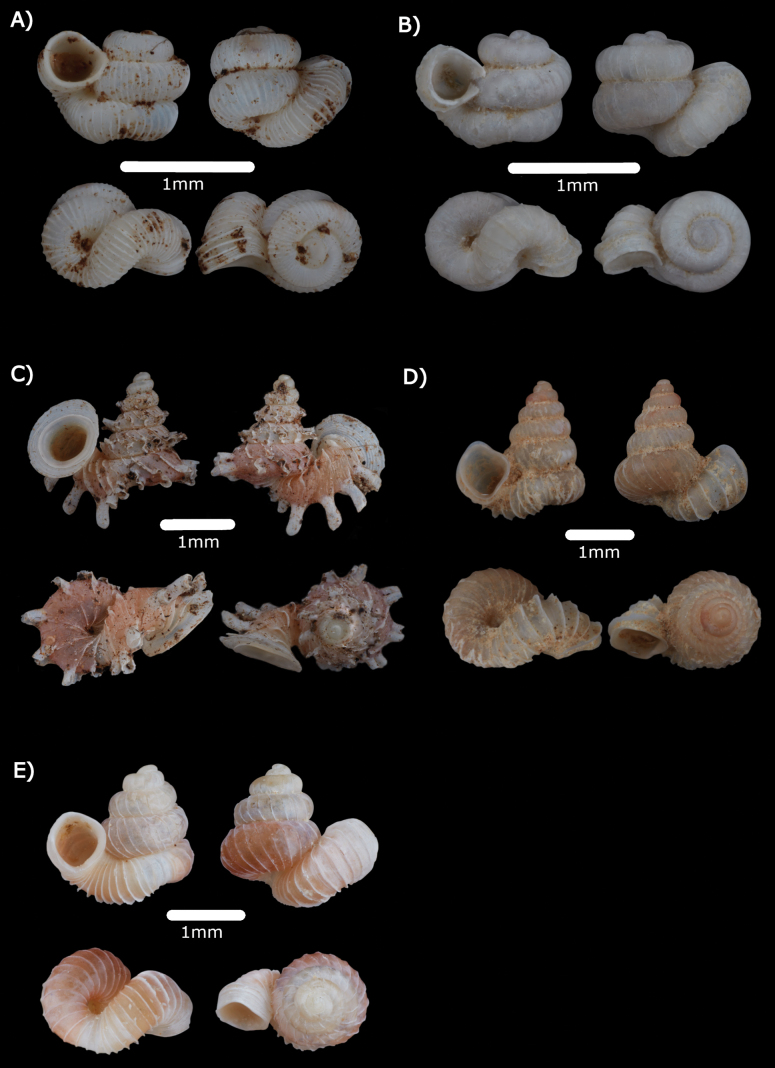
**A***Opisthostomabrachyacrumlambii* Vermeulen, 1991 ME 14480 **B***Opisthostomatridens* Vermeulen, 1991 ME 14481 **C***Plectostomaanisopterum* (Vermeulen, 1994) ME 14479 **D***Plectostomaausteni* (E. A. Smith, 1894a) ME 14478 Gua Rumbang **E***Plectostomapumilio* (E. A. Smith, 1894a) ME 14477.

**Type locality.** “Sarawak 1^st^ Div.: W of Kpg. Lobang Batu 12.5 km S of Tebakang”.

**Material examined.** Malaysia • Sarawak, Padawan, Gua Rumbang; 1°16.77'N, 110°15.69'E; 2 Sep. 2022–28 Jun. 2023; N.S. Nasir, M.E Marzuki, J.Y. Lee, and M.Z Khalik leg.; ME 14480, ME 15027, MZU.MOL.22.211.

**Distribution.** Scattered localities between Bau and Serian-Padawan limestone hills in western Sarawak. Also found in Sabah. Endemic to Borneo (Marzuki et al. 2021).

**Remarks.** Living snails were found foraging in leaf-litter and plant debris at the base of limestone cliffs.

***Opisthostomatridens* Vermeulen, 1991**

Fig. 9B

**Type locality.** “Sarawak 1^st^ Div.: Kpg. Beratok along road Kuching-Serian”.

**Material examined.** Malaysia • Sarawak, Padawan, Gua Rumbang; 1°16.77'N, 110°15.69'E; 2 Sep. 2022–28 Jun. 2023; N.S. Nasir, M.E Marzuki, J.Y. Lee, and M.Z Khalik leg.; ME 14481, ME 15028, MZU.MOL.22.443.

**Distribution.** Scattered localities between Bau and Serian-Padawan limestone hills in western Sarawak. Endemic to western Sarawak region (Marzuki et al. 2021).

**Remarks.** Living snails found on wet vertical limestone surfaces covered with mosses.

***Plectostomaanisopterum* (Vermeulen, 1994)**

Figs 9C, 20D

**Type locality.** “G. Saak 1 mile W. of Begu, 24 miles S. of Kuching”.

**Material examined.** Malaysia • Sarawak, Padawan, Gua Rumbang; 1°16.77'N, 110°15.69'E; 2 Apr. 2016–28 Jun. 2023; N.S. Nasir, M.E Marzuki, J.Y. Lee, and M.Z Khalik leg.; ME 14479, ME 14987, ME 15026, MZU.MOL.16.112, MZU.MOL.22.134, MZU.MOL.23.142.

**Distribution.** Scattered localities in Serian-Padawan limestone hills in western Sarawak. Endemic to western Sarawak (Vermeulen 1994).

**Remarks.** Living snails found on wet vertical limestone surfaces covered with mosses. It can also be found among boulders.

***Plectostomaausteni* (E. A. Smith, 1894a)**

Figs 9D, 21B

**Type locality.** “Rumbang, Sarawak”.

**Material examined.** Malaysia • Sarawak, Padawan, Gua Rumbang; 1°16.77'N, 110°15.69'E; 2 Apr. 2016–28 Jun. 2023; N.S. Nasir, M.E Marzuki, J.Y. Lee, and M.Z Khalik leg.; ME 14478, ME 14986, ME 15025, MZU.MOL.22.144, MZU.MOL.16.113.

**Distribution.** Scattered localities between Bau and Serian-Padawan limestone hills in western Sarawak. Endemic to western Sarawak region (Marzuki et al. 2021).

**Remarks.** Living snails found on wet vertical limestone surfaces and was observed foraging inside the rock crevices and cave walls, avoiding direct exposure to light.

***Plectostomapumilio* (E. A Smith, 1894a)**

Figs 9E, 21A

**Type locality.** “Rumbang, Sarawak”.

**Material examined.** Malaysia • Sarawak, Padawan, Gua Rumbang; 1°16.77'N, 110°15.69'E; 2 Apr. 2016–28 Jun. 2023; N.S. Nasir, M.E Marzuki, J.Y. Lee, and M.Z Khalik leg.; ME 14477, ME 14985, ME 15024, MZU.MOL.16.114.

**Distribution.** Scattered localities in Serian-Padawan limestone hills in western Sarawak. Endemic to western Sarawak region (Vermeulen 1994).

**Remarks.** Living snails found on wet vertical limestone surfaces covered with mosses.

**Subclass Heterobranchia Burmeister, 1837**

**Family Achatinidae Swainson, 1840**

***Allopeasclavulinum* (Potiez & Michaud, 1838)**

Fig. 10A

**Figure 10.**
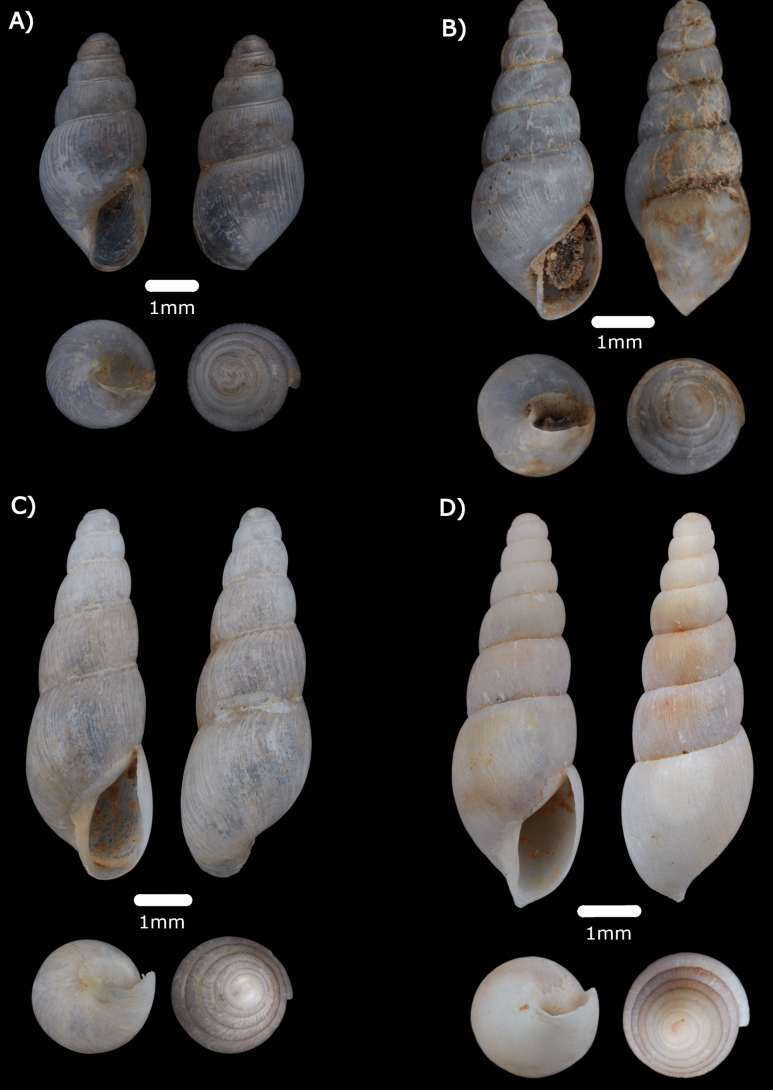
**A***Allopeasclavulinum* (Potiez & Michaud, 1838) ME 14485 **B***Allopeasgracile* (T. Hutton, 1834) ME 14486 **C***Opeashannense* (Rang, 1831) ME 14484 **D***Paropeasachatinaceum* (L. Pfeiffer, 1846) ME 14487.

**Type locality.** “L’ile Bourbon” [= La Réunion].

**Material examined.** Malaysia • Sarawak, Padawan, Gua Rumbang; 1°16.77'N, 110°15.69'E; 2 Sep. 2022–28 Jun. 2023; N.S. Nasir, M.E Marzuki, J.Y. Lee, and M.Z Khalik leg.; ME 14485, ME 14990, ME 15035, MZU.MOL.22.159.

**Distribution.** Widely distributed in Borneo. Circumtropical (Marzuki et al. 2021; Vermeulen and Liew 2022).

**Remarks.** Among leaf-litter and plant debris at the base of limestone cliffs. Only empty shells were found.

***Allopeasgracile* (T. Hutton, 1834)**

Fig. 10B

**Type locality.** “Mirzapoor, India”.

**Material examined.** Malaysia • Sarawak, Padawan, Gua Rumbang; 1°16.77'N, 110°15.69'E; 2 Sep. 2022–28 Jun. 2023; N.S. Nasir, M.E Marzuki, J.Y. Lee, and M.Z Khalik leg.; ME 14486, ME 14991, ME 15036, MZU.MOL.22.161.

**Distribution.** Widely distributed in Borneo. Circumtropical (Marzuki et al. 2021; Vermeulen and Liew 2022).

**Remarks.** Among leaf-litter and plant debris at the base of limestone cliffs. Only empty shells were found.

***Opeashannense* (Rang, 1831)**

Fig. 10C

**Type locality.** “Village of Hann, Cap Vert peninsula, Dakar, Senegal”.

**Material examined.** Malaysia • Sarawak, Padawan, Gua Rumbang; 1°16.77'N, 110°15.69'E; 2 Sep. 2022–28 Jun. 2023; N.S. Nasir, M.E Marzuki, J.Y. Lee, and M.Z Khalik leg.; ME 14484, ME 14989, ME 15034, MZU.MOL.22.158.

**Distribution.** Scattered localities between Bau and Serian-Padawan limestone hills in western Sarawak. Also found in Sabah. Distributed from Central America to Africa and Pacific (Marzuki et al. 2021; Vermeulen and Liew 2022).

**Remarks.** Among leaf-litter and plant debris at the base of limestone cliffs. Only empty shells were found.

***Paropeasachatinaceum* (L. Pfeiffer, 1846)**

Fig. 10D

**Type locality.** “Java”.

**Material examined.** Malaysia • Sarawak, Padawan, Gua Rumbang; 1°16.77'N, 110°15.69'E; 2 Apr. 2016–28 Jun. 2023; N.S. Nasir, M.E Marzuki, J.Y. Lee, and M.Z Khalik leg.; ME 14487, ME 14992, ME 15037, MZU.MOL.16.147, MZU.MOL.22.160.

**Distribution.** Widely distributed in Borneo. Distributed from South to East Asia, South-east Asia, and Pacific Islands (Marzuki et al. 2021; Vermeulen and Liew 2022).

**Remarks.** Among leaf-litter and plant debris at the base of limestone cliffs. Only empty shells were found.

**Family Achatinellidae Gulick, 1873**

***Elasmiassundanum* (Möllendorff, 1897)**

Fig. 11A

**Figure 11.**
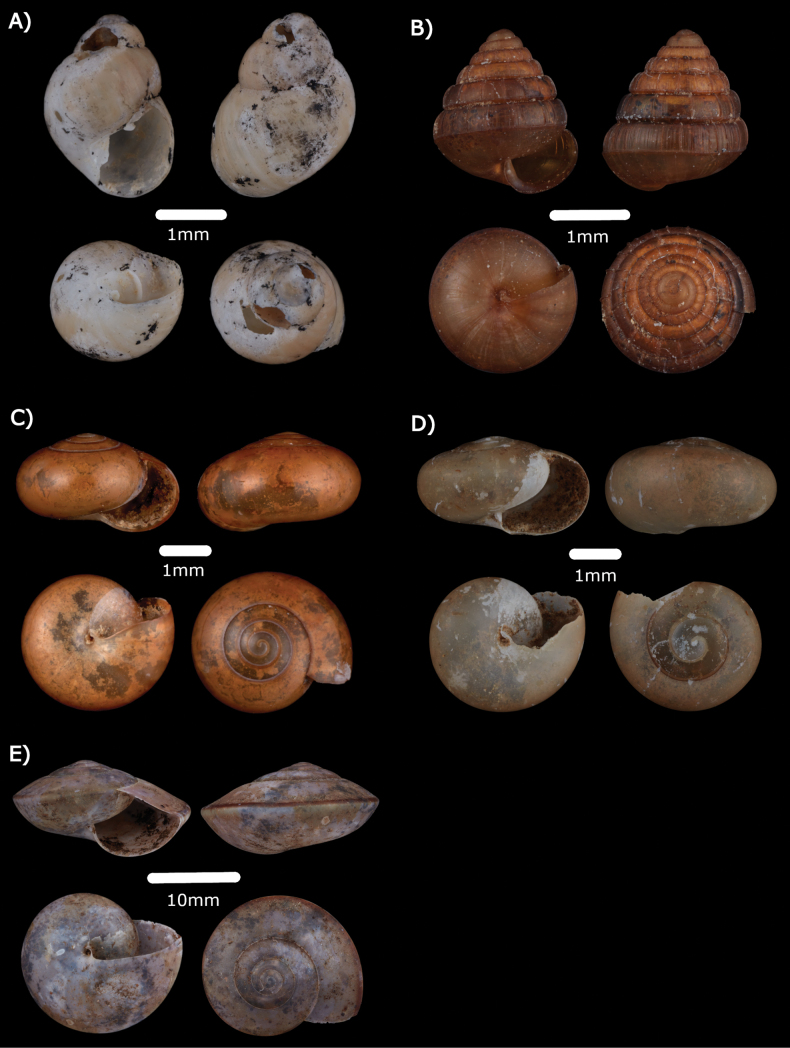
**A***Elasmiassundanum* (Möllendorff, 1897) ME 15058 **B***Rahulararicostulata* (E. A. Smith, 1893) MZU.MOL.22.139 **C***Macrochlamyssanctijohni* (Godwin-Austen, 1891) ME 14500 **D** Semi adult *Macrochlamysinfans* (Reeve, 1854) ME 14501 **E***Vitrinulaglutinosa* (Metcalfe, 1852) ME 14502.

**Type locality.** “Java”.

**Material examined.** Malaysia • Sarawak, Padawan, Gua Rumbang; 1°16.77'N, 110°15.69'E; 2 Sep. 2022–28 Jun. 2023; N.S. Nasir, M.E Marzuki, J.Y. Lee, and M.Z Khalik leg.; ME 14514, ME 15058, MZU.MOL.23.149.

**Distribution.** Scattered localities between Bau and Serian-Padawan limestone hills in western Sarawak to Niah at further northern Sarawak (Marzuki et al. 2021). Also found in Sumatra and Java (van Benthem-Jutting 1952).

**Remarks.** Among leaf-litter and plant debris at the base of limestone cliffs. Only empty shells were found.

**Family Ariophantidae Godwin-Austen, 1883**

***Rahulararicostulata* (E. A. Smith, 1893)**

Figs 11B, 21C

**Type locality.** “Busau or Busan, Sarawak”.

**Material examined.** Malaysia • Sarawak, Padawan, Gua Rumbang; 1°16.77'N, 110°15.69'E; 2 Sep. 2022–28 Jun. 2023; N.S. Nasir, M.E Marzuki, J.Y. Lee, and M.Z Khalik leg.; ME 14495, ME 15043, MZU.MOL.22.139.

**Distribution.** Scattered localities between Bau and Serian-Padawan limestone hills in western Sarawak. Endemic to western Sarawak (Marzuki et al. 2021).

**Remarks.** Living snails were observed foraging on leaf surface of trees at the base of limestone cliffs.

***Macrochlamyssanctijohni* (Godwin-Austen, 1891)**

Fig. 11C

**Type locality.** “Busan Hills”.

**Material examined.** Malaysia • Sarawak, Padawan, Gua Rumbang; 1°16.77'N, 110°15.69'E; 2 Sep. 2022–28 Jun. 2023; N.S. Nasir, M.E Marzuki, J.Y. Lee, and M.Z Khalik leg.; ME 14500, ME 14998, ME 15048, MZU.MOL.22.451.

**Distribution.** Scattered localities between Bau and Serian-Padawan limestone hills in western Sarawak to Niah in northern Sarawak (Marzuki et al. 2021). Also found in Palawan, Philippines (Smith 1895).

**Remarks.** Among leaf-litter and plant debris at the base of limestone cliffs. Only empty shells were found.

***Macrochlamysinfans* (Reeve, 1854)**

Figs 11D, 21D

**Type locality.** “Borneo”.

**Material examined.** Malaysia • Sarawak, Padawan, Gua Rumbang; 1°16.77'N, 110°15.69'E; 2 Apr. 2016–28 Jun. 2023; N.S. Nasir, M.E Marzuki, J.Y. Lee, and M.Z Khalik leg.; ME 14501, ME 15061, MZU.MOL.16.118, MZU.MOL.22.449, MZU.MOL.23.144.

**Distribution.** Widely distributed in Borneo. Distributed from Sumatra to Philippines (Vermeulen and Liew 2022).

**Remarks.** Empty shells were found among leaf-litter and plant debris at the base of limestone cliffs while living snails were found foraging on leaf surfaces.

***Vitrinulaglutinosa* (Metcalfe, 1852)**

Figs 11E, 22A

**Type locality.** “Borneo”.

**Material examined.** Malaysia • Sarawak, Padawan, Gua Rumbang; 1°16.77'N, 110°15.69'E; 2 Sep. 2022–28 Jun. 2023; N.S. Nasir, M.E Marzuki, J.Y. Lee, and M.Z Khalik leg.; ME 14502, ME 15049, MZU.MOL.22.130, MZU.MOL.23.146.

**Distribution.** Scattered localities between Bau and Serian-Padawan limestone hills in western Sarawak to Mulu at northern Sarawak. Endemic to Borneo (Marzuki et al. 2021).

**Remarks.** Empty shells were found among leaf-litter and plant debris at the base of limestone cliffs while living snails were found foraging on leaf surfaces. Individuals of this species show variability in the height of the spire and in the colour (pale to dark brown) (Marzuki et al. 2021).

***Microcystinaarabii* Marzuki, Liew & Mohd-Azlan, 2021**

Fig. 12A

**Figure 12.**
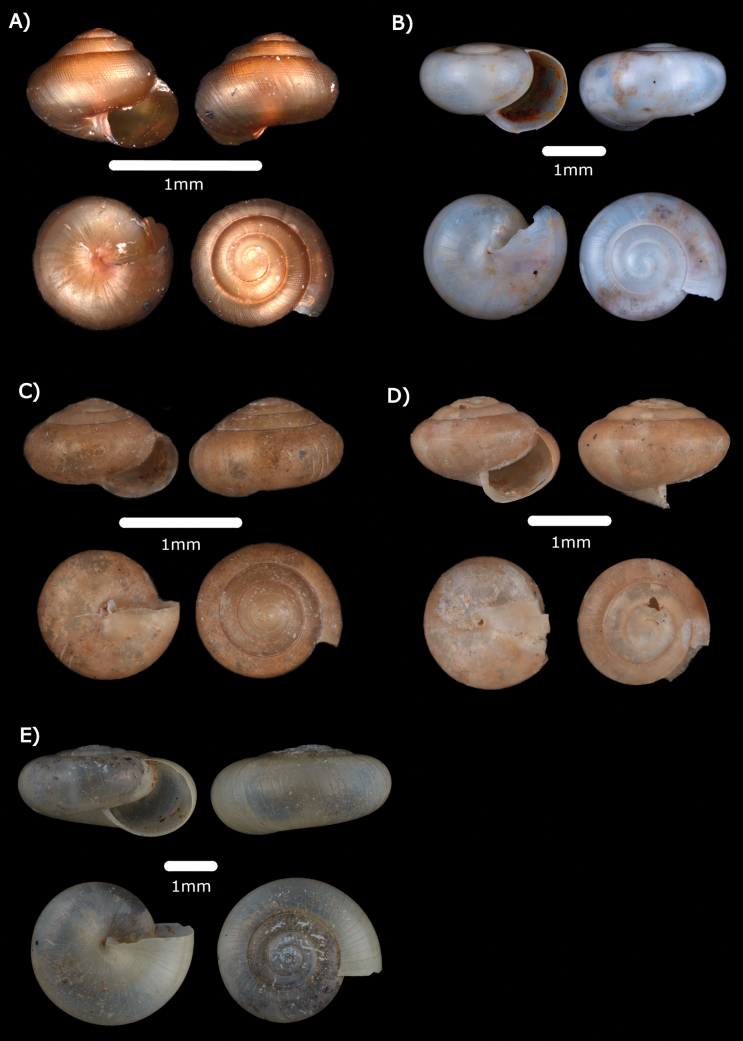
**A***Microcystinaarabii* Marzuki, Liew & Mohd-Azlan, 2021 ME 14505 **B***Microcystinakilat* Marzuki, Liew & Mohd-Azlan, 2021 ME 15050 **C***Microcystinaparipari* Marzuki, Liew & Mohd-Azlan, 2021 ME 15052 **D***Microcystinaphysotrochus* Vermeulen, Liew & Schilthuizen, 2015 ME 15060 **E***Microcystinavitreiformis* (Möllendorff, 1897) MZU.MOL.16.117.

**Type locality.** “Malaysia, Sarawak, Kuching, Division, Bukit Sokwang (Site 2), northern site of Gunung Doya, limestone hill along Skio road, 2.05 miles E Bau”.

**Material examined.** Malaysia • Sarawak, Padawan, Gua Rumbang; 1°16.77'N, 110°15.69'E; 2 Sep. 2022–28 Jun. 2023; N.S. Nasir, M.E Marzuki, J.Y. Lee, and M.Z Khalik leg.; ME 14505, MZU.MOL.23.152.

**Distribution.** Scattered localities between Bau and Serian-Padawan limestone hills in western Sarawak. Endemic to western Sarawak (Marzuki et al. 2021).

**Remarks.** Among leaf-litter and plant debris at the base of limestone cliffs. Only empty shells were found.

***Microcystinakilat* Marzuki, Liew & Mohd-Azlan, 2021**

Fig. 12B

**Type locality.** “Malaysia, Sarawak, Kuching Division, Lobang Angin (Site 2), limestone outcrop near Sungai Sarawak Kanan, 1.75 miles W Bau”.

**Material examined.** Malaysia • Sarawak, Padawan, Gua Rumbang; 1°16.77'N, 110°15.69'E; 28 Jun. 2023; N.S. Nasir, M.E Marzuki, J.Y. Lee, and M.Z Khalik leg.; ME 15050, MZU.MOL.23.151.

**Distribution.** Scattered localities between Bau and Serian-Padawan limestone hills in western Sarawak. Endemic to western Sarawak (Marzuki et al. 2021).

**Remarks.** Among leaf-litter and plant debris at the base of limestone cliffs. Only empty shells were found.

***Microcystinaparipari* Marzuki, Liew & Mohd-Azlan, 2021**

Fig. 12C

**Type locality.** “Malaysia, Sarawak, Kuching Division, Fairy Cave (Site 2), south part of Gunung Kapor, 4 miles SW Bau”.

**Material examined.** Malaysia • Sarawak, Padawan, Gua Rumbang; 1°16.77'N, 110°15.69'E; 2 Sep. 2022–28 Jun. 2023; N.S. Nasir, M.E Marzuki, J.Y. Lee, and M.Z Khalik leg.; ME 14503, ME 15000, ME 15052, MZU.MOL.22.132.

**Distribution.** Scattered localities between Bau and Serian-Padawan limestone hills in western Sarawak. Endemic to western Sarawak (Marzuki et al. 2021).

**Remarks.** Among leaf-litter and plant debris at the base of limestone cliffs. Only empty shells were found.

***Microcystinaphysotrochus* Vermeulen, Liew & Schilthuizen, 2015**

Fig. 12D

**Type locality.** “Malaysia, Sabah, Sandakan Province, Kinabatangan Valley, Batu Keruak 2, near Sukau”.

**Material examined.** Malaysia • Sarawak, Padawan, Gua Rumbang; 1°16.77'N, 110°15.69'E; 28 Jun. 2023; N.S. Nasir, M.E Marzuki, J.Y. Lee, and M.Z Khalik leg.; ME 15060.

**Distribution.** Scattered localities between Bau and Serian-Padawan limestone hills in western Sarawak to Niah in northern Sarawak. Also found in Sabah. Endemic to Borneo (Marzuki et al. 2021; Vermeulen and Liew 2022).

**Remarks.** Among leaf-litter and plant debris at the base of limestone cliffs. Only empty shells were found.

***Microcystinavitreiformis* (Möllendorff, 1897)**

Fig. 12E

**Type locality.** “Java”.

**Material examined.** Malaysia • Sarawak, Padawan, Gua Rumbang; 1°16.77'N, 110°15.69'E; 2 Apr. 2016–28 Jun. 2023; N.S. Nasir, M.E Marzuki, J.Y. Lee, and M.Z Khalik leg.; ME 15051, MZU.MOL.16.117.

**Distribution.** Scattered localities between Bau and Serian-Padawan limestone hills in western Sarawak to Niah in northern Sarawak. Also found in Java and its adjacent islands, Indonesia (Nurinsiyah, 2021).

**Remarks.** Only empty shells were found.

**Family Camaenidae Pilsbry, 1895**

***Amphidromusangulatus* Fulton, 1896**

Fig. 13A

**Figure 13.**
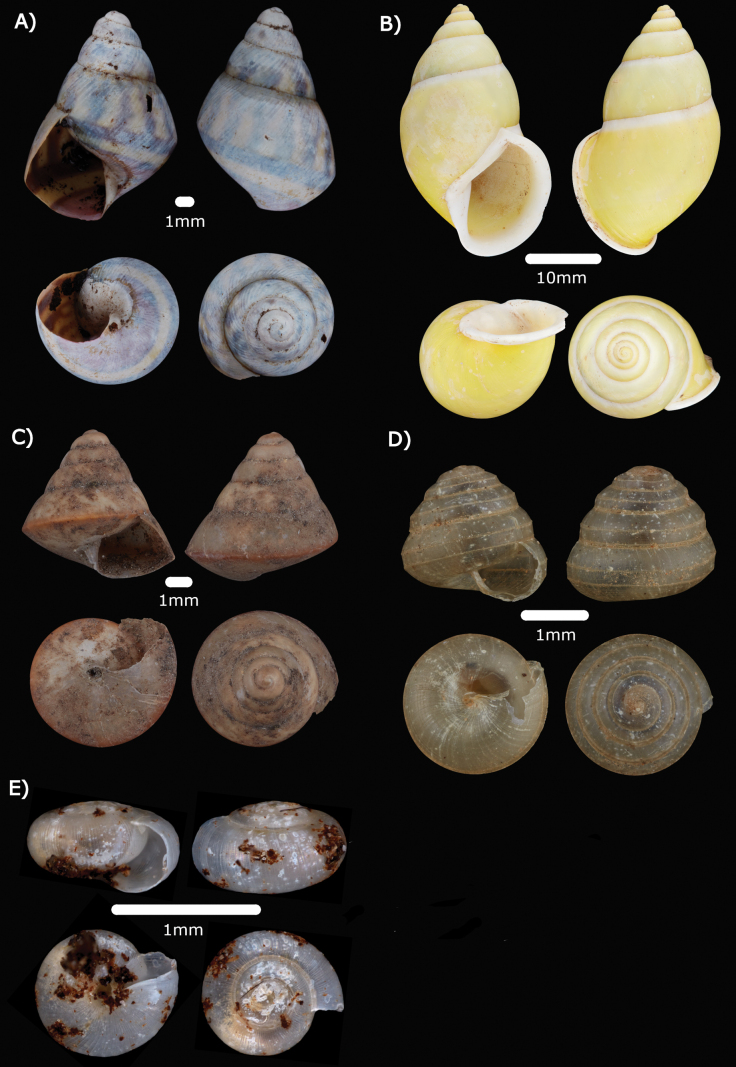
**A***Amphidromusangulatus* Fulton, 1896 MZU.MOL.22.147 **B***Amphidromusepidemiae* Wang, 2021 ME 14507 **C***Ganesellaacris* (Benson, 1859) MZU.MOL.22.150 **D***Philalankakusana* (Aldrich, 1889) MZU.MOL.16.111 **E***Sundacharopaargos* Vermeulen & Liew, 2022 ME 14511.

**Type locality.** “Sarawak”.

**Material examined.** Malaysia • Sarawak, Padawan, Gua Rumbang; 1°16.77'N, 110°15.69'E; 2 Sep. 2022; N.S. Nasir, M.E Marzuki, J.Y. Lee, and M.Z Khalik leg.; MZU.MOL.22.147.

**Distribution.** Scattered localities between Bau and Serian-Padawan limestone hills in western Sarawak, to Niah, further northern Sarawak. Also found in West Kalimantan (Marzuki et al. 2021).

**Remarks.** Living snails were observed foraging on leaf surface of trees at the base of limestone cliffs.

***Amphidromusepidemiae* Wang, 2021**

Fig. 13B

**Type locality.** “Sarawak, Kuching Division, Bau”.

**Material examined.** Malaysia • Sarawak, Padawan, Gua Rumbang; 1°16.77'N, 110°15.69'E; 2 Sep. 2022–28 Jun. 2023; ME 14507, ME 15004, ME 15054, MZU.MOL.22.183.

**Distribution.** Scattered localities between Bau and Serian-Padawan limestone hills in western Sarawak. Endemic to western Sarawak.

**Remarks.** Among leaf-litter and plant debris at the base of limestone cliffs. Only empty shells were found.

***Ganesellaacris* (Benson, 1859)**

Fig. 13C

**Type locality.** “Teria Ghát montium Khasiæ” [= Khasi Hills, Teria Ghat, India].

**Material examined.** Malaysia • Sarawak, Padawan, Gua Rumbang; 1°16.77'N, 110°15.69'E; 2 Apr. 2016–2 Sep. 2022; N.S. Nasir, M.E Marzuki, J.Y. Lee, and M.Z Khalik leg.; ME 14508, MZU.MOL.22.150, MZU.MOL.16.146.

**Distribution.** Widely distributed with scattered localities in Borneo. Distributed from Sumatra to Java Indonesia, and South to Southeast Asian mainland (Marzuki et al. 2021; Vermeulen and Liew 2022).

**Remarks.** Among leaf-litter and plant debris at the base of limestone cliffs. Only empty shells were found.

**Family Charopidae Hutton, 1884**

***Philalankakusana* (Aldrich, 1889)**

Fig. 13D

**Type locality.** “Kusan and Penggiron districts in South-eastern Borneo”.

**Material examined.** Malaysia • Sarawak, Padawan, Gua Rumbang; 1°16.77'N, 110°15.69'E; 2 Apr. 2016–2 Sep. 2022; N.S. Nasir, M.E Marzuki, J.Y. Lee, and M.Z Khalik leg.; ME 14509, MZU.MOL.16.111.

**Distribution.** Widely distributed in Borneo. Distributed from West Malaysia to Papua (Marzuki et al. 2021; Vermeulen and Liew 2022).

**Remarks.** Among leaf-litter and plant debris at the base of limestone cliffs. Only empty shells were found.

***Sundacharopaargos* Vermeulen & Liew, 2022**

Fig. 13E

**Type locality.** “Malaysia, Sabah, upper Padas River valley, Long Pa Sia”.

**Material examined.** Malaysia • Sarawak, Padawan, Gua Rumbang; 1°16.77'N, 110°15.69'E; 2 Sep. 2022; N.S. Nasir, M.E Marzuki, J.Y. Lee, and M.Z Khalik leg.; ME 14511.

**Distribution.** Widely distributed in Borneo. Endemic to Borneo (Vermeulen and Liew 2022).

**Remarks.** Among leaf-litter and plant debris at the base of limestone cliffs. Only empty shells were found.

**Family Chronidae Thiele, 1931**

***Kaliellabarrakporensis* (Reeve, 1852)**

Figs 14A, 22B

**Figure 14.**
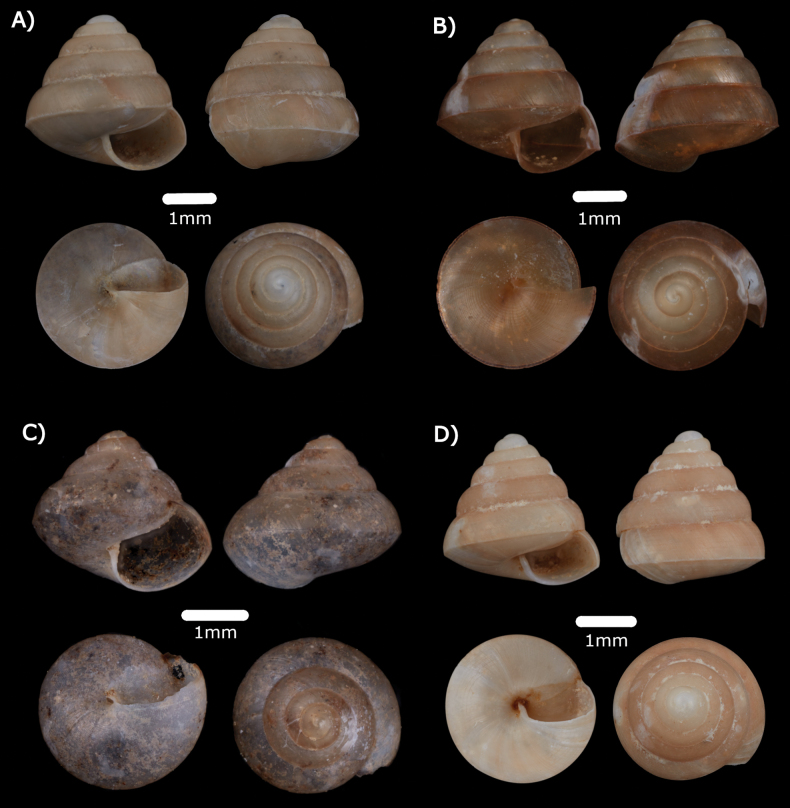
**A***Kaliellabarrakporensis* (Reeve, 1852) ME 14489 **B***Kaliellabusauensis* (E. A. Smith, 1895) ME 14488 **C***Kaliellacalculosa* (Gould, 1852) ME 14493 **D***Kaliellamicroconus* (Mousson, 1865) ME 14490.

**Type locality.** “Barrakpore Indiae (Bacon)” [= Barrackpore, West Bengal, India].

**Material examined.** Malaysia • Sarawak, Padawan, Gua Rumbang; 1°16.77'N, 110°15.69'E; 2 Sep. 2022–28 Jun. 2023; N.S. Nasir, M.E Marzuki, J.Y. Lee, and M.Z Khalik leg.; ME 14489, ME 14994, ME 15039, MZU.MOL.22.148, MZU.MOL.23.143.

**Distribution.** Widely distributed in Borneo. Distributed from Africa and South Asia mainland to Indo-Australian archipelago and Europe (Marzuki et al. 2021; Vermeulen and Liew 2022).

**Remarks.** Living snails were observed foraging on leaf surface of trees at the base of limestone cliffs.

***Kaliellabusauensis* (E. A. Smith, 1895)**

Figs 14B, 22C

**Type locality.** “Busau, Sarawak”.

**Material examined.** Malaysia • Sarawak, Padawan, Gua Rumbang; 1°16.77'N, 110°15.69'E; 2 Apr. 2016–28 Jun. 2023; N.S. Nasir, M.E Marzuki, J.Y. Lee, and M.Z Khalik leg.; ME 14488, ME 14993, ME 15038, MZU.MOL.16.115, MZU.MOL.22.144.

**Distribution.** Scattered localities between Bau and Serian-Padawan limestone hills in western Sarawak. Endemic to western Sarawak.

**Remarks.** Living snails were observed foraging on leaf surface of trees at the base of limestone cliffs. Vermeulen and Liew (2022) mentioned that this species is synonymous with *K.barrakporensis*. In contrast, Marzuki et al. (2021) listed it as separate species. *Kaliellabusauensis* has a higher, dark brown shell with a cancellated shell surface caused by prominent spiral grooves and oblique radial riblets.

***Kaliellacalculosa* (Gould, 1852)**

Fig. 14C

**Type locality.** “Tahiti” [= Tahiti Island, French Polynesia].

**Material examined.** Malaysia • Sarawak, Padawan, Gua Rumbang; 1°16.77'N, 110°15.69'E; 2 Sep. 2022; N.S. Nasir, M.E Marzuki, J.Y. Lee, and M.Z Khalik leg.; ME 14493, ME 14506, MZU.MOL.22.450.

**Distribution.** Widely distributed in Borneo. Distributed from India to Australia and Pacific (Marzuki et al. 2021; Vermeulen and Liew 2022).

**Remarks.** Living snails were observed foraging on leaf surface of trees at the base of limestone cliffs.

***Kaliellamicroconus* (Mousson, 1865)**

Fig. 14D

**Type locality.** “Lomma-Lomma (Viti)” [= Loma Loma, Fiji].

**Material examined.** Malaysia • Sarawak, Padawan, Gua Rumbang; 1°16.77'N, 110°15.69'E; 2 Sep. 2022–28 Jun. 2023; N.S. Nasir, M.E Marzuki, J.Y. Lee, and M.Z Khalik leg.; ME 14490, ME 14995, ME 15040, MZU.MOL.22.168.

**Distribution.** Widely distributed in Borneo. Distributed from South-east Asia to Australia and the Pacific Islands (Marzuki et al. 2021; Vermeulen and Whitten 1998).

**Remarks.** Living snails were observed foraging on leaf surface of trees at the base of limestone cliffs.

***Kaliellarumbangensis* (E. A. Smith, 1895)**

Figs 15A, 22D

**Figure 15.**
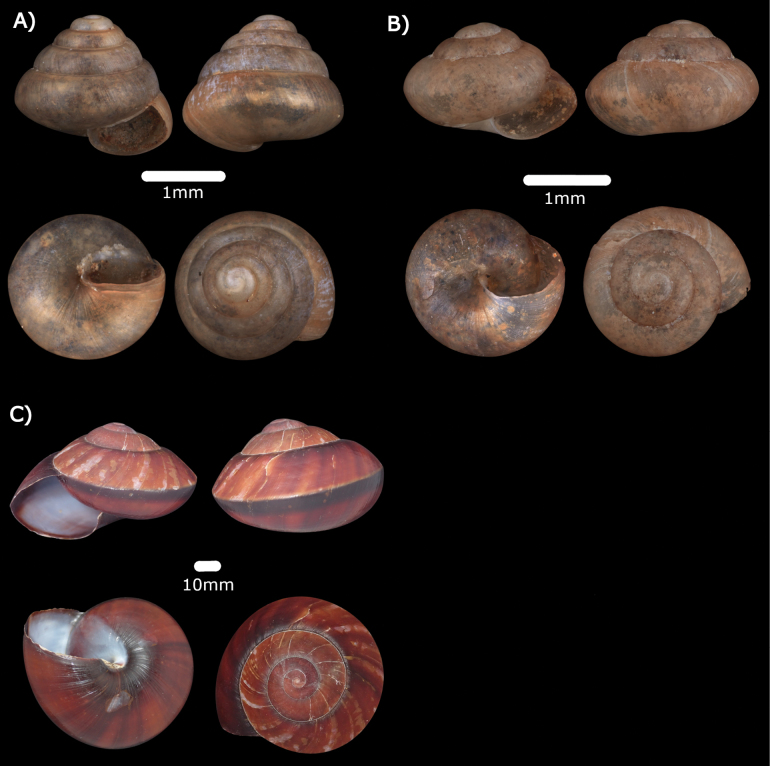
**A***Kaliellarumbangensis* (E. A. Smith, 1895) ME 14491 **B***Kaliellascandens* (Cox, 1872) ME 14492 **C***Exrhysotabrookei* (A. Adams & Reeve, 1850) MZU.MOL.22.151.

**Type locality.** “Rumbang, Sarawak and Mount Rabong”.

**Material examined.** Malaysia • Sarawak, Padawan, Gua Rumbang; 1°16.77'N, 110°15.69'E; 2 Sep. 2022–28 Jun. 2023; N.S. Nasir, M.E Marzuki, J.Y. Lee, and M.Z Khalik leg.; ME 14491, ME 14504, ME 14996, ME 15041, MZU.MOL.22.163, MZU.MOL.22.145.

**Distribution.** Padawan limestone hills in western Sarawak only. Endemic to western Sarawak (Smith, 1895).

**Remarks.** Living snails were observed foraging on leaf surface of trees at the base of limestone cliffs. Vermeulen and Liew (2022) listed this as synonymous with *K.barrakporensis*. *Kaliellarumbangensis* differs from *K.barrakporensis* by its smaller size and by the inconspicuous (or even absent) peripheral keel on the last whorl.

***Kaliellascandens* (Cox, 1872)**

Fig. 15B

**Type locality.** “Port Macquarie, east coast of Australia”.

**Material examined.** Malaysia • Sarawak, Padawan, Gua Rumbang; 1°16.77'N, 110°15.69'E; 2 Sep. 2022–28 Jun. 2023; N.S. Nasir, M.E Marzuki, J.Y. Lee, and M.Z Khalik leg.; ME 14492, ME 14997, ME 15042, MZU.MOL.22.444.

**Distribution.** Widely distributed in Borneo. Distributed from South-east Asia to Australia and the Pacific Islands (Marzuki et al. 2021; Vermeulen et al. 2015).

**Remarks.** Living snails were observed foraging on leaf surface of trees at the base of limestone cliffs.

***Exrhysotabrookei* (A. Adams & Reeve, 1850)**

Figs 15C, 23

**Type locality.** “Mountains of Borneo”.

**Material examined.** Malaysia • Sarawak, Padawan, Gua Rumbang; 1°16.77'N, 110°15.69'E; 2 Sep. 2022; N.S. Nasir, M.E Marzuki, J.Y. Lee, and M.Z Khalik leg.; MZU.MOL.22.151.

**Distribution.** Widespread in Borneo. Endemic to Borneo (Marzuki et al. 2021).

**Remarks.** Living snails were found foraging in limestone crevices and in leaf litter. This is the largest native land snail species in Borneo.

**Family Diapheridae Panha & Naggs, 2010**

***Platycochliumsarawakense* Laidlaw, 1950**

Figs 16A, 24A

**Figure 16.**
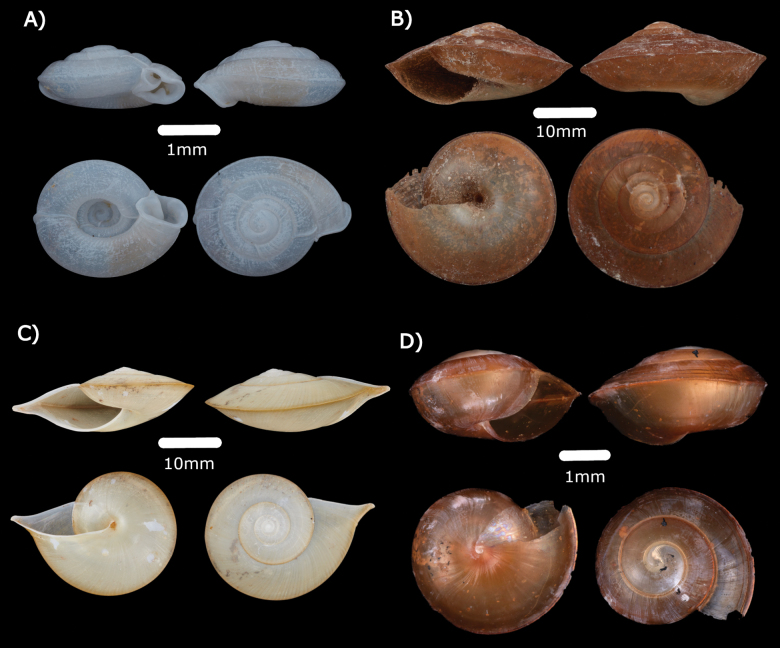
**A***Platycochliumsarawakense* Laidlaw, 1950 ME 14483 **B***Dyakiasubdebilis* E. A. Smith, 1895 ME 14496 **C***Rhinocochlisnasuta* (Metcalfe, 1852) ME 14493 **D** Juvenile *Geotrochusconicoides* (Metcalfe, 1852) ME 15057.

**Type locality.** “Gunong Kapor, Bau District, Sarawak”.

**Material examined.** Malaysia • Sarawak, Padawan, Gua Rumbang; 1°16.77'N, 110°15.69'E; 2 Sep. 2022; N.S. Nasir, M.E Marzuki, J.Y. Lee, and M.Z Khalik leg.; ME 14483, ME 14988, ME 15033, MZU.MOL.22.153.

**Distribution.** Scattered localities between Bau and Serian-Padawan limestone hills in western Sarawak. Endemic to western Sarawak (Marzuki et al. 2021).

**Remarks.** Living snails found foraging among leaf-litter and plant debris at the base of limestone cliffs.

**Family Dyakiidae Gude & B. B. Woodward, 1921**

***Dyakiasubdebilis* E. A. Smith, 1895**

Figs 16B, 24B

**Type locality.** “Sarawak”.

**Material examined.** Malaysia • Sarawak, Padawan, Gua Rumbang; 1°16.77'N, 110°15.69'E; 2 Sep. 2022–28 Jun. 2023; N.S. Nasir, M.E Marzuki, J.Y. Lee, and M.Z Khalik leg.; ME 14496, ME 15044, MZU.MOL.22.141, MZU.MOL.23.136.

**Distribution.** Scattered localities between Bau and Serian-Padawan limestone hills in western Sarawak. Endemic to western Sarawak (Marzuki et al. 2021).

**Remarks.** Living snails were found foraging on leaf surface of trees at the base of limestone cliffs and on limestone surfaces covered with mosses and lichens.

***Rhinocochlisnasuta* (Metcalfe, 1852)**

Fig. 16C

**Type locality.** “Borneo”.

**Material examined.** Malaysia • Sarawak, Padawan, Gua Rumbang; 1°16.77'N, 110°15.69'E; 2 Sep. 2022–28 Jun. 2023; N.S. Nasir, M.E Marzuki, J.Y. Lee, and M.Z Khalik leg.; ME 14497, ME 15002, ME 15045, MZU.MOL.22.146.

**Distribution.** Widely distributed in Borneo. Endemic to Borneo (Marzuki et al. 2021).

**Remarks.** Living snails were observed foraging on leaf surface of trees at the base of limestone cliffs.

**Family Geotrochidae Schileyko, 2002**

***Geotrochusconicoides* (Metcalfe, 1852)**

Fig. 16D

**Type locality.** “Borneo”.

**Material examined.** Malaysia • Sarawak, Padawan, Gua Rumbang; 1°16.77'N, 110°15.69'E; 28 Jun. 2023; N.S. Nasir, M.E Marzuki, J.Y. Lee, and M.Z Khalik leg.; ME 15057, MZU.MOL.23.150

**Distribution.** Widely distributed in Borneo. Widespread. Also found in Sumatra, Indonesia and Palawan, Philippines (Marzuki et al. 2021; Vermeulen and Liew 2022).

**Remarks.** Among leaf-litter and plant debris at the base of limestone cliffs. Only empty shells were found.

**Family Helicarionidae Bourguignat, 1877**

***Helicariondyakanum* (Godwin-Austen, 1891)**

Figs 17A, 24C

**Figure 17.**
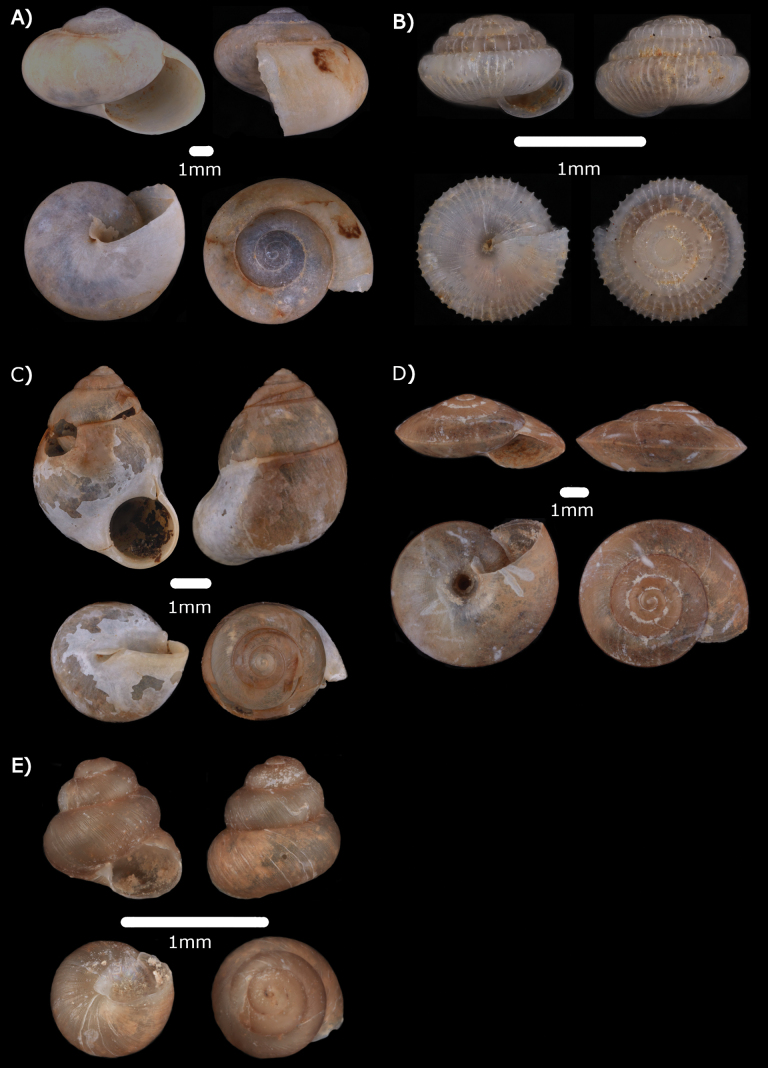
**A***Helicariondyakanum* (Godwin-Austen, 1891) ME 14499 **B***Paralaomasarawakensis* Marzuki, Liew & Mohd-Azlan, 2021 ME 14510 **C***Pupinaevansi* Godwin-Austen, 1889 ME 15029 **D***Videnanepiadelphos* Vermeulen & Liew, 2022 ME 14513 **E***Pupisomadioscoricola* (C. B. Adams, 1845) ME 14498.

**Type locality.** “Busan Hills, Borneo” [= Jambusan Hills, Bau, Sarawak].

**Material examined.** Malaysia • Sarawak, Padawan, Gua Rumbang; 1°16.77'N, 110°15.69'E; 2 Sep. 2022–28 Jun. 2023; N.S. Nasir, M.E Marzuki, J.Y. Lee, and M.Z Khalik leg.; ME 14499, ME 15001.

**Distribution.** Widely distributed in Sarawak. In Sabah on Mount Trusmadi only. Endemic to Borneo (Marzuki et al. 2021; Vermeulen and Liew 2022).

**Remarks.** Living snails were observed foraging on leaf surface of trees at the base of limestone cliffs.

**Family Punctidae Morse, 1864**

***Paralaomasarawakensis* Marzuki, Liew & Mohd-Azlan, 2021**

Figs 17B, 24D

**Type locality.** “Limestone hill along Skio road, 2.05 miles E Bau, Northern site of Gua Doya, Bukit Sokwang (Site 3), Kuching Division, Sarawak, Malaysia”.

**Material examined.** Malaysia • Sarawak, Padawan, Gua Rumbang; 1°16.77'N, 110°15.69'E; 2 Sep. 2022–28 Jun. 2023; N.S. Nasir, M.E Marzuki, J.Y. Lee, and M.Z Khalik leg.; ME 14510, ME 15055, MZU.MOL.22.448.

**Distribution.** Scattered localities between Bau and Serian-Padawan limestone hills in western Sarawak. Also found in Baram, in northern Sarawak. Endemic to Sarawak (Marzuki et al. 2021).

**Remarks.** Living snails were found foraging among leaf-litter and plant debris at the base of limestone cliffs.

**Family Pupinidae L. Pfeiffer, 1853**

***Pupinaevansi* Godwin-Austen, 1889**

Fig. 17C

**Type locality.** “From deposit in Cave A, Borneo” [= Tupak Cave, Jambusan Hills, see Cranbrook 2013)].

**Material examined.** Malaysia • Sarawak, Padawan, Gua Rumbang; 1°16.77'N, 110°15.69'E; 2 Sep. 2022–28 Jun. 2023; N.S. Nasir, M.E Marzuki, J.Y. Lee, and M.Z Khalik leg.; ME 15029.

**Distribution.** Scattered localities in Serian-Padawan limestone hills in western Sarawak (Marzuki et al. 2021). Also found in Sirhassen, Natuna Island (Smith 1894b).

**Remarks.** Among leaf-litter and plant debris at the base of limestone cliffs. Only empty shells were found.

**Family Trochomorphidae Möllendorff, 1890**

***Videnanepiadelphos* Vermeulen & Liew, 2022**

Fig. 17D

**Type locality.** “Danum valley Conservation Area, Tawau Prov., Sabah”.

**Material examined.** Malaysia • Sarawak, Padawan, Gua Rumbang; 1°16.77'N, 110°15.69'E; 2 Sep. 2022–28 Jun. 2023; N.S. Nasir, M.E Marzuki, J.Y. Lee, and M.Z Khalik leg.; ME 14513, ME 15056, MZU.MOL.22.447.

**Distribution.** Widely distributed in Borneo. Widespread. Also found in Panaitan island, Indonesia (Vermeulen and Liew 2022)

**Remarks.** Only empty shells were found among leaf-litter and plant debris at the base of limestone cliffs.

**Family Valloniidae Morse, 1864**

***Pupisomadioscoricola* (C. B. Adams, 1845)**

Fig. 17E

**Type locality.** “Jamaica”.

**Material examined.** Malaysia • Sarawak, Padawan, Gua Rumbang; 1°16.77'N, 110°15.69'E; 2 Sep. 2022–28 Jun. 2023; N.S. Nasir, M.E Marzuki, J.Y. Lee, and M.Z Khalik leg.; ME 14498, ME 15003, ME 15046, MZU.MOL.22.445.

**Distribution.** Widely distributed in Borneo. Widespread. Distributed in Africa, Asia, Australia, and America (Marzuki et al. 2021).

**Remarks.** Living snails were observed foraging on leaf surface of trees at the base of limestone cliffs.

**Subclass Neritimorpha Golikov & Starobogatov, 1975**

**Family Hydrocenidae Troschel, 1857**

***Georissaeveretti* E. A. Smith, 1895**

Fig. 18A

**Figure 18.**
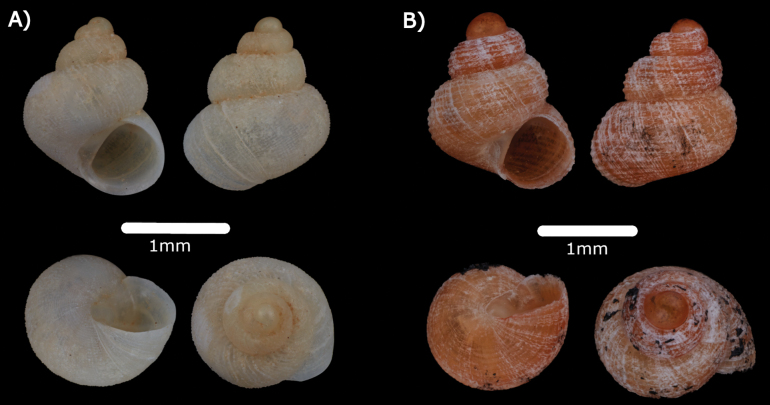
**A***Georissaeveretti* E. A. Smith, 1895 ME 14482 **B***Georissahungerfordi* Godwin-Austen, 1889 ME 15032.

**Type locality.** “Rumbang, W. Sarawak”.

**Material examined.** Malaysia • Sarawak, Padawan, Gua Rumbang; 1°16.77'N, 110°15.69'E; 2 Sep. 2022; N.S. Nasir, M.E Marzuki, J.Y. Lee, and M.Z Khalik leg.; ME 14482, MZU.MOL.22.157.

**Distribution.** Scattered localities between Bau and Serian-Padawan limestone hills in western Sarawak to Niah in northern Sarawak. Also found in Sabah, Sepulut valley. Widespread. Endemic to Borneo (Khalik et al. 2019; Marzuki et al. 2021).

**Remarks.** Living snails were observed foraging on wet limestone wall surfaces covered with mosses.

***Georissahungerfordi* Godwin-Austen, 1889**

Fig. 18B

**Type locality.** “Borneo”.

**Material examined.** Malaysia • Sarawak, Padawan, Gua Rumbang; 1°16.77'N, 110°15.69'E; 28 Jun. 2023; N.S. Nasir, M.E Marzuki, J.Y. Lee, and M.Z Khalik leg.; ME 15032.

**Distribution.** Scattered localities between Bau and Serian-Padawan limestone hills in western Sarawak. Endemic to western Sarawak (Khalik et al. 2019; Marzuki et al. 2021).

**Remarks.** Living snails were observed foraging on wet limestone wall surfaces covered with mosses.

**Figure 19.**
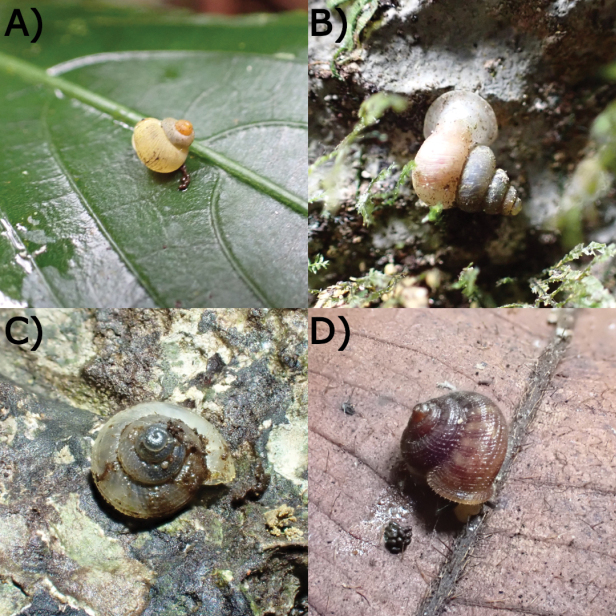
Living snails from Gua Rumbang. **A***Pincernaglobosa* (H. Adams, 1871) MZU.MOL.22.135 **B***Stomacosmethishosei* (Godwin-Austen, 1889) MZU.MOL.22.136 **C***Craspedotropisborneensis* (Godwin-Austen, 1889) MZU.MOL.23.141 **D***Japoniaborneensis* (E. A. Smith, 1893) MZU. MOL.23.138. Images not to scale.

**Figure 20.**
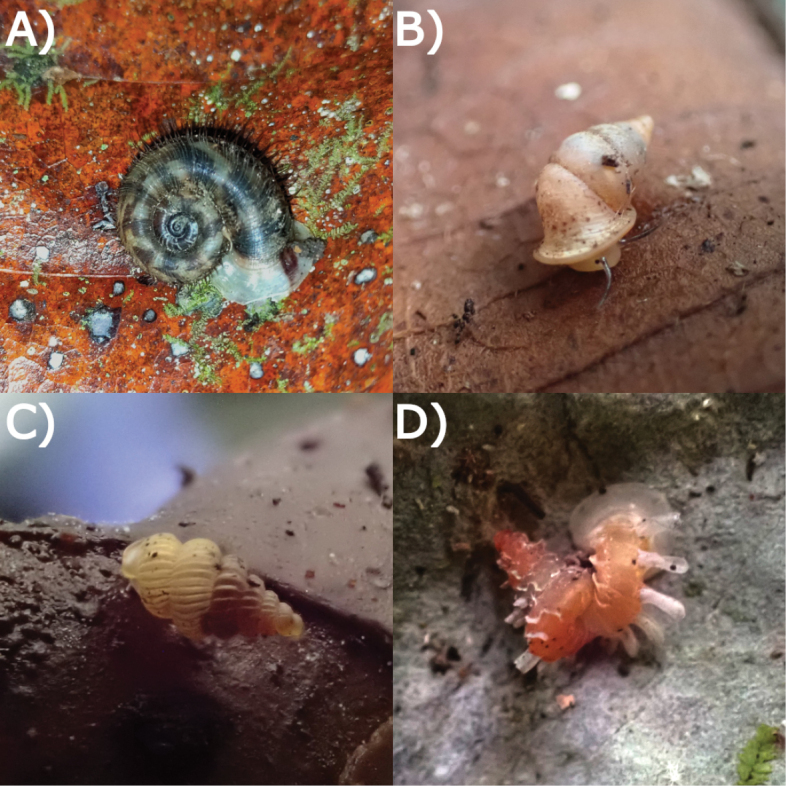
Living snails from Gua Rumbang. **A***Opisthoporusbiciliatus* (Mousson, 1849) ME 14979 **B***Diplommatinaadversa* (H. Adams & A. Adams, 1851) MZU. MOL.23.140 **C***Diplommatinabaritensis* E. A. Smith, 1893 MZU. MOL.23.137 **D***Plectostomaanisopterum*ME 14479 (Vermeulen, 1994). All images not to scale.

**Figure 21.**
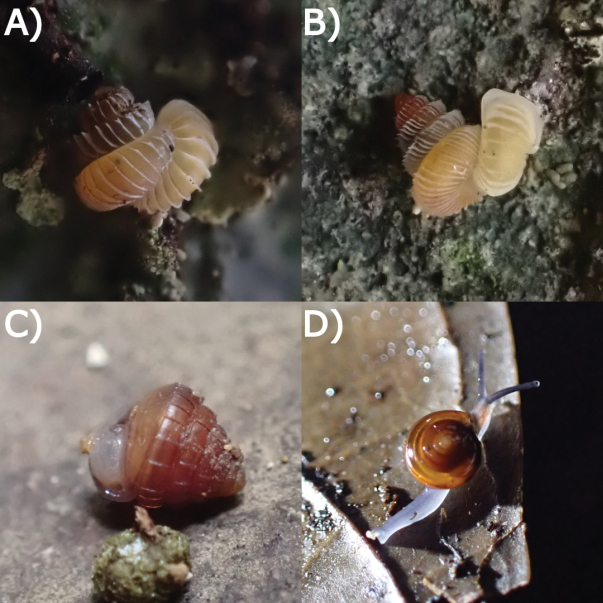
Living snails from Gua Rumbang. **A***Plectostomapumilio* (E. A. Smith, 1894a) ME 14477 **B***Plectostomaausteni* (E. A. Smith, 1894a) ME 14477 **C***Rahulararicostulata* (E. A. Smith, 1893) MZU.MOL.22.139 **D** Semi adult *Macrochlamysinfans* (Reeve, 1854) MZU.MOL.23.144. Images not to scale.

**Figure 22.**
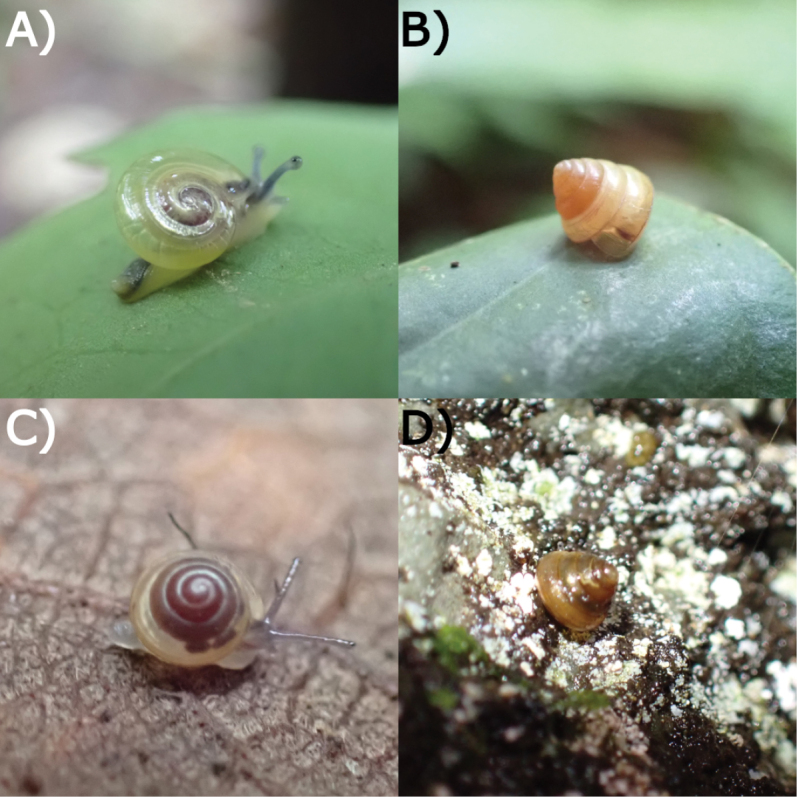
Living snails from Gua Rumbang. **A** Juvenile *Vitrinulaglutinosa* (Metcalfe, 1852) MZU.MOL.23.146 **B***Kaliellabarrakporensis* (Reeve, 1852) MZU.MOL.23.143 **C***Kaliellabusauensis* (E. A. Smith, 1895) MZU.MOL.22.144 **D***Kaliellarumbangensis* (E. A. Smith, 1895) MZU.MOL.22.163. Images not to scale.

**Figure 23.**
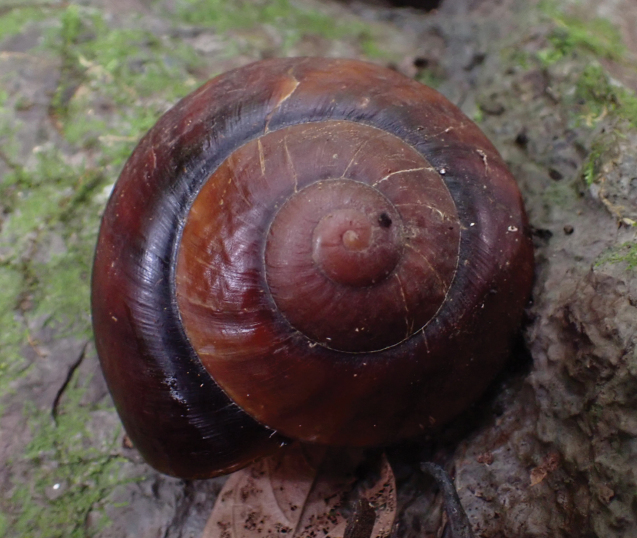
Living snail from Gua Rumbang. *Exrhysotabrookei* (A. Adams & Reeve, 1850) MZU.MOL.22.151.

**Figure 24.**
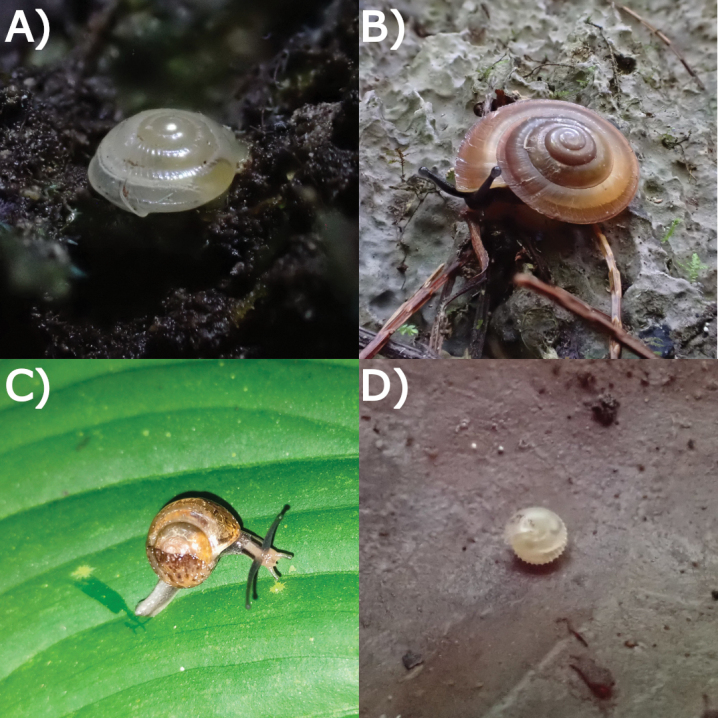
Living snails from Gua Rumbang. **A***Platycochliumsarawakense* Laidlaw, 1950 MZU.MOL.22.153 **B***Dyakiasubdebilis* E. A. Smith, 1895 MZU.MOL.23.136 **C***Helicariondyakanum* (Godwin-Austen, 1891) ME 14499 **D***Paralaomasarawakensis* Marzuki, Liew & Mohd-Azlan, 2021 ME 14510. All imaged not to scale.

## Supplementary Material

XML Treatment for Diplommatina rumbangensis

## ﻿Additional information

### Author ORCIDs

Jie Ying Lee https://orcid.org/0009-0002-6223-3814

Mohd Zacaery Khalik https://orcid.org/0000-0001-7098-0743

### Data availability

All of the data that support the findings of this study are available in the main text or Supplementary Information.

## Supplementary materials

Supplementary material 1 List of land snails and their microhabitat preferences of Gua Rumbang Data type xlsx https://binary.pensoft.net/file/1034264This dataset is made available under the Open Database License (http://opendatacommons.org/licenses/odbl/1.0/). The Open Database License (ODbL) is a license agreement intended to allow users to freely share, modify, and use this Dataset while maintaining this same freedom for others, provided that the original source and author(s) are credited.Author: Nurul Syafiqah Nasir
